# Vitamin E‐Related Molecular Signatures in Adolescent Idiopathic Scoliosis: A Hypothesis‐Generating Multi‐Omics Analysis

**DOI:** 10.1002/fsn3.72190

**Published:** 2026-08-06

**Authors:** Ying Ma, Yuankun Han, Zhizhong Geng, Sitong Fang, Jun Ren, Shoujian Wang, Tianxiang He, Lingjun Kong, Min Fang

**Affiliations:** ^1^ Shuguang Hospital Shanghai University of Traditional Chinese Medicine Shanghai China; ^2^ Institute of Tuina Shanghai Institute of Traditional Chinese Medicine Shanghai China

**Keywords:** adolescent idiopathic scoliosis, oxidative stress, prostaglandin signaling, tocopherol, vascular response, vitamin E

## Abstract

Adolescent idiopathic scoliosis (AIS) involves systemic bone‐metabolic dysregulation and paraspinal microenvironment remodeling, but whether nutrition‐related micronutrient‐associated molecular programs overlap with these alterations remains unclear. Vitamin E (VE), comprising lipid‐soluble tocopherols and tocotrienols, is linked to membrane protection, lipid peroxidation control, inflammatory mediator regulation, and endothelial responses. This study examined whether VE‐related molecular signatures converge on AIS‐associated redox, lipid‐inflammatory, vascular, and multicellular remodeling programs. Peripheral blood miRNA data (GSE235203) and bone marrow transcriptomic data (GSE110359) were integrated using HERB‐based compound mapping, VE‐AIS shared‐target enrichment, feature prioritization, intradisease GSEA, single‐cell localization, CellChat analysis, and NHANES contextualization. HERB mapping generated a VE/tocopherol‐related prioritization signal, not direct evidence of VE involvement in AIS. Forty‐two shared VE‐AIS targets were enriched mainly in oxidative stress, glutathione/peroxidase activity, and glutathione metabolism, with additional lipid‐inflammatory and vascular signals. Five prioritized genes (SOD1, GCLC, PTGS1, PTGS2, and KDR) defined redox‐buffering, lipid‐inflammatory, and vascular‐response axes. Single‐cell and CellChat analyses localized these signatures mainly to endothelial, dendritic/APC‐like, MSC‐like, and stromal/osteogenic populations, suggesting a predicted concave‐side enrichment of inflammatory‐endothelial‐stromal communication. Structural analyses supported the computational plausibility of α‐tocopherol compatibility with selected proteins, particularly PTGS2. No AIS cohort with measured VE exposure or status was analyzed. NHANES provided external clinical nutrition context rather than AIS‐specific validation. Overall, VE‐related signatures overlapped with redox, lipid‐inflammatory, vascular‐response, and multicellular remodeling programs in AIS. These findings generate testable molecular hypotheses but do not show that VE intake, tocopherol status, or supplementation modifies AIS risk, severity, or progression.

## Graphical abstract

Integrative analysis of plasma miRNA data (GSE235203) and BM‐MSC transcriptomic data (GSE110359), combined with database‐derived VE/tocopherol target mapping, prioritized SOD1, GCLC, PTGS1, PTGS2, and KDR. Single‐cell localization, CellChat analysis, structural simulation, NHANES contextualization, and GSE286204 supported hypothesis‐generating links to redox/glutathione, lipid‐inflammatory, vascular, bone‐remodeling, and multicellular communication programs. These computational findings do not establish that vitamin E intake, tocopherol status, or supplementation modifies AIS risk, severity, or progression.

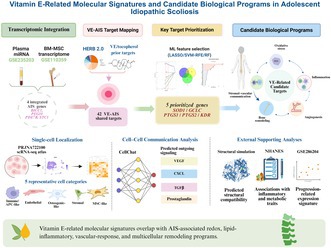

## 1 Introduction

Adolescent idiopathic scoliosis (AIS) is the most common three‐dimensional spinal deformity in adolescents, affecting approximately 1%–4% and showing female predominance (Dunn et al. 2018; Konieczny et al. 2013). Although spinal deformity defines AIS clinically, the condition is increasingly seen as a systemic process of aberrant tissue remodeling involving bone metabolism, skeletal muscle function, extracellular matrix remodeling, oxidative stress, and tissue microenvironmental regulation (Pumputis et al. 2025). Circulating signals, bone marrow osteogenic programs, and local musculoskeletal tissues may contribute to adaptive and maladaptive changes.

Redox imbalance and lipid‐driven inflammatory remodeling are increasingly implicated in AIS. Bone mass decreases (Yang et al. 2023), impaired osteogenic differentiation of AIS bone marrow mesenchymal stem cells (BM‐MSCs) (Park et al. 2009), and dysregulated circulating miRNA have also been linked to altered bone metabolism and disease severity (Chen et al. 2022). At the local paraspinal muscle level, concave‐convex asymmetry has been observed across molecular phenotype (Chen et al. 2022; Park et al. 2009), inflammatory signaling (Shao et al. 2023), extracellular matrix remodeling (Sun et al. 2026), and reactive oxygen species (ROS)‐associated stress responses (Li et al. 2026). These observations point to oxidative stress and lipid‐mediated inflammatory remodeling as plausible contributors to tissue dysfunction in AIS.

Vitamin E (VE), a family of lipid‐soluble tocopherols and tocotrienols, may provide a useful entry point for examining AIS from a nutritional systems‐biology perspective. As a diet‐derived micronutrient, VE is obtained primarily from plant oils, nuts, seeds, whole grains, and green leafy vegetables (Zaaboul and Liu 2022). Its biological availability is shaped by dietary fat intake, intestinal lipid absorption, and circulating lipoprotein transport (Casati et al. 2020). Because VE is lipid‐soluble, its nutritional interpretation requires attention not only to dietary intake but also to lipid‐dependent absorption, lipoprotein transport, and lipid‐adjusted circulating tocopherol status. Beyond its classical antioxidant role, VE participates in the regulation of lipid peroxidation, inflammatory mediator synthesis, endothelial signaling, and stress‐response pathways, including NF‐κB, PI3K/Akt/mTOR, and MAPK signaling (Ungurianu et al. 2021). Tocopherols have also been reported to preserve BM‐MSC viability, attenuate ferroptosis‐related lipid oxidative injury, and support osteogenic differentiation through redox‐sensitive signaling pathways (Casati et al. 2020; Lan et al. 2022). Importantly, VE isoforms are not functionally interchangeable, as tocopherols and tocotrienols differ in antioxidant capacity, lipid‐mediator regulation, and anti‐inflammatory activity (Es‐Sai et al. 2025). Because AIS‐related tissue remodeling is increasingly discussed in the context of oxidative imbalance, lipid inflammation, and endothelial‐stromal adaptation, VE therefore provides a biologically plausible lens for exploring whether lipid‐phase antioxidant, inflammatory, and endothelial‐response programs overlap with AIS‐relevant microenvironmental remodeling. It is therefore considered here as a nutritional molecular entry point for hypothesis generation rather than as a proven modifier of AIS.

Despite these observations, it remains unclear whether VE‐associated molecular signatures are linked to AIS through coherent cross‐tissue mechanisms or whether they merely reflect isolated correlative changes. In particular, circulating molecular abnormalities, bone marrow transcriptomic alterations, and local tissue multicellular remodeling have rarely been integrated into a single analytical framework. To address this gap, this study integrates peripheral blood miRNA profiles, bone marrow transcriptomics, VE‐AIS shared‐target analysis, machine‐learning‐based feature prioritization, single‐cell transcriptomic localization, CellChat analysis, and structural simulation of α‐tocopherol‐target compatibility.

One important limitation of database‐derived nutrient‐target studies is that molecular mapping alone does not establish measured nutritional exposure or circulating nutrient status. This distinction is especially relevant for vitamin E, since serum tocopherol concentrations are influenced by lipid transport and may not directly reflect dietary intake. Ideally, AIS‐specific validation would combine dietary vitamin E intake, serum α‐tocopherol and γ‐tocopherol, lipid profiles, Cobb angle, BMD, BMI, pubertal maturity, curve progression, and oxidative or inflammatory biomarkers. However, no publicly available AIS cohort currently combines these variables. Therefore, NHANES was incorporated only as an external adolescent clinical nutrition context to examine vitamin E exposure and status in relation to skeletal, metabolic, pubertal, and inflammatory variables available in the general adolescent population. The NHANES analysis was not intended to validate AIS‐specific mechanisms. Because multiple adolescent endpoints were examined, NHANES associations were interpreted as nominal and hypothesis‐generating unless supported after correction for multiple testing. An exploratory AIS‐progression‐related miRNA analysis was also included in the Data [Link], [Link], [Link], [Link], [Link], [Link], [Link], [Link], [Link], [Link] to provide molecular progression context, but not as vitamin E‐specific validation.

This study therefore aimed to identify hypothesis‐generating, database‐derived VE‐related molecular signatures that overlap with AIS‐associated redox, lipid‐inflammatory, vascular‐response, and multicellular remodeling programs, and to interpret these findings within a broader adolescent nutrition context without implying AIS‐specific nutritional validation.

## 2 Materials and Methods

### 2.1 Data Sources

**FIGURE 1:**
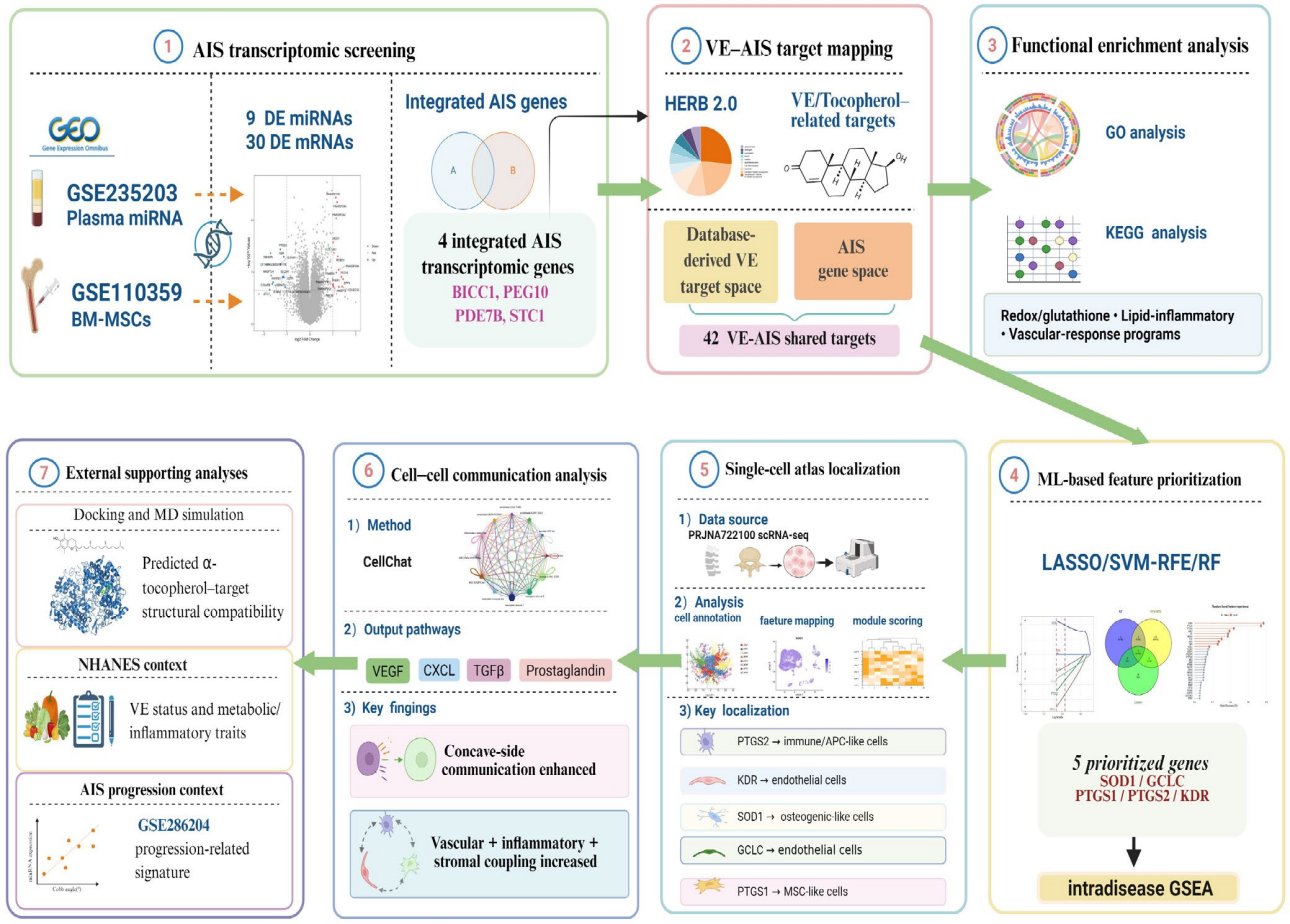
Overall workflow of the hypothesis‐generating multi‐omics analysis. Peripheral blood miRNA data (GSE235203) and bone marrow transcriptomic data (GSE110359) were integrated to identify overlapping candidate targets. HERB‐based compound mapping was used as a compound‐prioritization step, followed by VE‐AIS shared‐target enrichment, feature‐gene prioritization, intradisease GSEA, single‐cell localization, CellChat‐based communication analysis, structural simulation, and NHANES‐based adolescent clinical nutrition contextualization.

Representative peripheral blood miRNA expression profiles and bone marrow transcriptomic datasets for adolescent idiopathic scoliosis (AIS) were retrieved from the Gene Expression Omnibus (GEO) database. These AIS molecular datasets did not include measured dietary vitamin E intake, serum tocopherol status, lipid‐adjusted tocopherol concentrations, or supplementation exposure. GSE235203 was used to identify AIS‐associated circulating miRNA abnormalities, whereas GSE110359 characterized AIS‐related transcriptional alterations in the bone marrow compartment. The workflow comprised differential‐expression analysis, target overlap, HERB‐based compound prioritization, VE‐AIS shared‐target enrichment, complementary feature prioritization, exploratory intradisease pathway stratification, single‐cell localization, CellChat analysis, structural simulation, and NHANES‐based external adolescent clinical nutrition contextualization. Detailed dataset information is summarized in Table 1, and the workflow is shown in Figure 1 (Yuan et al. 2024; Zhuang et al. 2019).

**TABLE 1:** Summary of the GEO datasets included in this study.

GEO accession	GSE235203	GSE110359
Disease	AIS	AIS
Specimen	Peripheral blood plasma exosomes	Bone marrow‐derived mesenchymal stem cells
Molecular layer	miRNA expression profile	mRNA/transcriptome expression profile
Platform	Illumina HiSeq 2500	Affymetrix Human Transcriptome Array 2.0
Sample size	AIS = 6, normal = 6	AIS = 12, control = 5

### 2.2 Differential Expression Analysis, Functional Annotation, and Target Prediction

Processed expression matrices and platform annotation files were retrieved from GEO and analyzed in R. Where applicable, values were log2‐transformed, probe identifiers were mapped to official gene symbols, and duplicate gene symbols were collapsed. Differential expression was assessed between AIS and control samples in GSE235203 and GSE110359. Differentially expressed miRNAs in GSE235203 were identified using |log_2_FC| > 2 and FDR < 0.05, whereas mRNAs in GSE110359 were screened using |log_2_FC| > 1 and nominal *p* < 0.05. Because of the small dataset of GSE110359, the mRNA threshold was treated as exploratory and carried forward for downstream biological prioritization rather than confirmatory biomarker discovery. Volcano plots and heatmaps were used for visualization, and complete differential‐expression tables were retained in the Data [Link], [Link], [Link], [Link], [Link], [Link], [Link], [Link], [Link], [Link].

Differentially expressed miRNAs were imported into FunRich 3.1.3 (Fonseka et al. 2021) for Gene Ontology (GO) functional annotation, transcription factor enrichment analysis, and miRNA target prediction using the software's built‐in target annotation resources. Predicted targets were standardized to official gene symbols, duplicate entries were removed, and the resulting candidate target gene set was used for integrative analysis with bone marrow differentially expressed mRNAs.

### 2.3 Identification of Overlapping Targets and HERB‐Based Compound Mapping

Predicted target genes of the differentially expressed miRNAs were intersected with the differentially expressed mRNAs from GSE110359 to identify overlapping candidate targets. The four overlapping genes were then submitted to HERB 2.0 for compound annotation. Compound occurrence frequency was used only as a hypothesis‐generating prioritization signal and was not interpreted as a quantitative evidence score for compound‐target strength, biological activity, or disease relevance. Because four‐gene compound mapping cannot establish causal compound‐disease relationships, nutritional exposure, or direct VE involvement in AIS, the VE‐related signal was subsequently evaluated within a broader VE‐AIS shared‐target enrichment framework and downstream biological analyses.

### 2.4 Acquisition of VE‐ and AIS‐Related Targets and Enrichment Analysis of Shared Genes

To construct a VE‐associated target set, the exact search terms “vitamin E,” “α‐tocopherol,” “γ‐tocopherol,” and “tocopherol” were queried in SwissTargetPrediction, DrugBank, TTD, STITCH, and ClinPGx. For SwissTargetPrediction, targets with Probability > 0 were retained to maximize sensitivity; this permissive threshold was interpreted as target‐set construction rather than high‐confidence target validation. AIS‐related disease targets were obtained from GeneCards, OMIM, MalaCards, and PharmGKB, with a GeneCards relevance score cutoff of ≥ 1. All source‐specific targets were exported with database labels, converted to official gene symbols, merged, and de‐duplicated before intersection analysis. The shared targets between VE and AIS were then identified and subjected to GO and KEGG enrichment analyses using DAVID. For multiple‐testing control, enrichment terms supported by DAVID FDR or Benjamini‐adjusted q values < 0.05 were emphasized as the primary enrichment results, whereas terms supported only by nominal *p* < 0.05 were labeled as contextual or exploratory signals and were not interpreted as confirmatory pathway evidence. Nominal *p* values, Benjamini‐adjusted q values, and FDR values exported from DAVID were retained in the Data [Link], [Link], [Link], [Link], [Link], [Link], [Link], [Link], [Link], [Link] (Sherman et al. 2022).

### 2.5 Prioritization of VE‐AIS Shared Genes Using Complementary Feature‐Selection Approaches

Using the normalized GSE110359 matrix, VE‐AIS shared genes were extracted as candidate features. To prioritize biologically interpretable candidate nodes within the VE‐AIS shared target set, three complementary feature‐selection approaches—least absolute shrinkage and selection operator (LASSO) regression, support vector machine‐recursive feature elimination (SVM‐RFE), and random forest—were applied in parallel. LASSO used 10‐fold cross‐validation to determine the optimal penalty parameter and retained genes with nonzero coefficients. SVM‐RFE selected the subset with the lowest cross‐validation error, while random forest ranked candidate genes by variable importance. No independent validation cohort was available, and formal resampling‐based feature‐selection stability analysis was not performed. Cross‐method overlap was therefore interpreted only as a qualitative prioritization, and the resulting five‐gene set as a prioritized candidate panel for biological interpretation, not a validated diagnostic or predictive biomarker set.

### 2.6 Exploratory Evaluation of Prioritized Feature Genes

Prioritized‐gene expression differences in GSE110359 were evaluated using the Wilcoxon rank‐sum test. Receiver operating characteristic (ROC) curves and the area under the curve (AUC) were calculated only as within‐dataset exploratory consistency checks. Because GSE110359 was small and imbalanced, and because no independent test set or external validation cohort was available, these ROC analyses were not interpreted as diagnostic model validation.

An artificial neural network (ANN) model based on the prioritized genes was retained only as an exploratory internal sensitivity analysis in Data S3. It was not used for feature discovery or main conclusions. Because feature prioritization and ANN evaluation used the same discovery dataset, the ANN output and AUC were treated as descriptive apparent performance susceptible to overfitting, optimism, and small‐sample instability, not validated prediction.

### 2.7 GSEA of Pathways Associated With Key Feature Genes

To characterize the pathway context associated with each prioritized feature gene within AIS samples, AIS cases were stratified into high‐ and low‐expression groups according to the median expression of each gene. GSEA was then performed using the full transcriptomic matrix and KEGG gene sets as the reference collection. Normalized enrichment scores, nominal *p* values, and FDR *q* values were retained in Data S4. Pathways meeting nominal *p* < 0.05 were visualized only as exploratory intradisease enrichment signals, and nominal‐only GSEA findings were not interpreted as confirmatory pathway activation.

### 2.8 Single‐Cell Transcriptomic Analysis and Cell Annotation

The publicly available AIS single‐cell transcriptomic dataset PRJNA722100 from NCBI BioProject/SRA was analyzed to localize prioritized VE‐AIS feature genes within the paraspinal muscle microenvironment (Yang et al. 2021). This dataset contains four scRNA‐seq libraries (H66206_Cv, H66206_Cc, H01045_Cv, and H01045_Cc) derived from bilateral paraspinal muscle tissues of two AIS patients, corresponding to the convex and concave sides, and was generated using the Illumina NovaSeq 6000 platform. Seurat v5 was used for quality control, normalization, dimensionality reduction, clustering, and cell annotation. Low‐quality cells were filtered using the following thresholds: nFeature_RNA 300–6500, nCount_RNA 500–40,000, and mitochondrial gene proportion < 20% (Hao et al. 2024; Shao et al. 2023). Highly variable genes were then identified, followed by unsupervised clustering based on principal component analysis. UMAP and t‐SNE were used for visualization. Cluster marker genes were identified using FindAllMarkers, and cell types were annotated based on canonical marker genes and relevant literature.

### 2.9 Single‐Cell Functional Localization and Immune Feature Analysis

After cell annotation, the proportions of different cell types were calculated according to sample_id and region (concave/convex). The prioritized feature genes SOD1, PTGS1, PTGS2, KDR, and GCLC were then mapped onto the single‐cell atlas using Feature Plot, DotPlot, and violin plots. Transcriptional differences between concave and convex regions were further compared in relevant cell populations.

Gene sets representing oxidative stress, glutathione metabolism, arachidonic acid metabolism, VEGF/angiogenesis, and inflammatory responses were constructed, and Add Module Score was used to calculate module scores to assess the distribution of these biological programs across cell types and regions. In addition, based on the normalized GSE110359 expression matrix, single‐sample GSEA (ssGSEA) was performed to evaluate the relative enrichment of predefined immune cell signatures, and Spearman correlation analysis was used to assess associations between key feature genes and immune features.

Module‐score comparisons and Spearman correlations were interpreted as descriptive localization and association analyses, not as donor‐independent validation of cell‐type‐specific pathway activity.

### 2.10 Cell–Cell Communication Analysis

To examine whether prioritized VE‐AIS functional programs were embedded within predicted multicellular communication networks, CellChat was applied to annotated cell populations relevant to inflammatory, vascular, stromal, and osteogenic remodeling. These included dendritic/APC‐like cells, inflammatory myeloid‐like cells, osteoclast/monocyte‐like cells, endothelial‐related cells, MSC‐like cells, and osteogenic stromal cells. Ligand‐receptor communication networks were constructed to infer predicted signaling pathways and sender‐receiver interactions. Communication analyses were performed separately for concave and convex regions. Because CellChat was applied to cells nested within donors, ligand‐receptor outputs were interpreted as descriptive predicted communication patterns rather than donor‐independent statistical evidence.

### 2.11 Molecular Docking and Molecular Dynamics Simulation

To assess potential structural interactions between a representative VE homolog and AIS‐associated key feature proteins, α‐tocopherol was selected for docking and MD simulations. This analysis was designed as a computational structural plausibility assessment and not as evidence of biological target engagement or functional modulation in AIS. KDR, PTGS1, PTGS2, and SOD1 were selected for molecular docking and MD simulation based on the availability of target‐matched protein structures and structural outputs. GCLC was retained as a transcriptomic redox/glutathione‐related feature gene but was not included in the primary docking/MD interpretation because MD output was not generated.

Protein structures were obtained from the Protein Data Bank and included human PTGS1/COX‐1 (PDB 6Y3C, chain A), human PTGS2/COX‐2 (PDB 5F19, chain A), human KDR/VEGFR2 kinase domain (PDB 5EW3, chain A), and a SOD1 domain II structural context, corresponding to the copper chaperone for SOD1 (PDB 6FN8, chain A). Detailed receptor regions, ligand information, docking grid centers, box sizes, docking assumptions, and MD settings are provided in Data S8.

α‐Tocopherol was chosen because it is the predominant circulating form of vitamin E in humans and provides a structurally tractable model, not because it is assumed to be the only relevant VE isoform in AIS. The two‐dimensional structure of α‐tocopherol was obtained from PubChem CID 1742129 and converted into a three‐dimensional structure for downstream analyses.

Protein structures were preprocessed using PyMOL (Liu et al. 2023) to remove water molecules and irrelevant small ligands. AutoDockTools was then used for hydrogen addition, charge assignment, and receptor/ligand preparation, followed by molecular docking using AutoDock Vina. The optimal docking pose was selected based on binding energy scores, and interaction patterns within the complexes were visualized using Discovery Studio 2019 and PyMOL. Available chain identifiers, grid settings, ligand protonation state, and residue numbering were recorded for reproducibility, and outputs without target‐matched receptor identity were excluded from target‐level interpretation.

Based on the optimal docking conformations, MD simulations were further carried out using GROMACS 2022. The AMBER14SB force field was used for proteins, GAFF2 parameters were applied to the ligand, and the system was solvated using the TIP3P water model with ion neutralization. Energy minimization, equilibration, and a 100 ns production run were performed at 310 K and 1 bar. After simulation, RMSD, RMSF, radius of gyration (Rg), solvent‐accessible surface area (SASA), and free energy landscape (FEL) were calculated. The MM/PBSA method was used to estimate binding free energies and the energetic contribution of key residues. Docking and MD results were interpreted as computational structural compatibility evidence and were not considered proof of biological target engagement, nutritional relevance, or functional regulation by VE in AIS tissues.

### 2.12 NHANES‐Based External Adolescent Clinical Nutrition Analysis

An external population‐based analysis was conducted using NHANES 2017–2018 data from participants aged 10–19 years, excluding those coded as pregnant and those with missing key exposure, outcome, or covariate data for each analysis. NHANES was incorporated only as external adolescent clinical nutrition context because AIS diagnosis, Cobb angle, curve type, Risser or Sanders stage, brace treatment, and longitudinal curve progression are not available in this dataset.

Serum vitamin E status was assessed using serum α‐tocopherol and γ‐tocopherol concentrations obtained from the NHANES Vitamin A, Vitamin E, and Carotenoids laboratory dataset. Because vitamin E is lipid‐soluble and transported through circulating lipoproteins, lipid‐adjusted tocopherol measures were used. Serum α‐tocopherol normalized to total cholesterol was defined as the primary vitamin E status variable and serum γ‐tocopherol normalized to total cholesterol as a secondary status variable.

Dietary vitamin E exposure was assessed using 2‐day mean α‐tocopherol intake where available. To account for differences in total energy intake, dietary vitamin E density was also calculated. Lumbar spine BMD was extracted from the NHANES DXA spine examination file, and BMI from the body measures file. High‐sensitivity C‐reactive protein was included as a marker of systemic inflammation. Among female participants, pubertal maturation was explored using age at menarche and post‐menarche status from the reproductive health questionnaire. Serum 25‐hydroxyvitamin D was included, where available, as a bone‐health covariate.

Weighted regression models assessed associations between vitamin E exposure or status variables and lumbar spine BMD, BMI, log‐transformed hs‐CRP, and menarche‐related measures. Models were adjusted for age, sex, race/ethnicity, BMI where appropriate, serum 25‐hydroxyvitamin D, and dietary fat or total energy intake in dietary models, while accounting for the NHANES weights, strata, and primary sampling units. Serum biomarker analyses incorporated NHANES examination weights, whereas dietary analyses used the corresponding 2‐day dietary weights. Endpoint‐specific analytic N, effect estimates, 95% confidence intervals, and nominal *p* values were designated for reporting. Because multiple exposures and endpoints were examined, NHANES associations were interpreted as nominal and hypothesis‐generating unless they remained supported after multiple‐testing correction, not as AIS‐specific validation.

### 2.13 Supplementary AIS Progression‐Related miRNA Contextual Analysis

An exploratory AIS progression‐related miRNA analysis was performed using GSE286204 to assess descriptive overlap between published progression‐associated circulating miRNA targets and the redox/glutathione, prostaglandin‐related, vascular‐response, and inflammatory modules identified in this study. Because GSE286204 lacks dietary VE intake, serum tocopherol, and lipid‐adjusted tocopherol variables, overlaps were interpreted as AIS progression‐related molecular context, not VE‐specific or progression‐validated evidence (Data S9).

## 3 Results

### 3.1 Differential miRNA and mRNA Expression Profiles Identify AIS‐Associated Molecular Abnormalities

**FIGURE 2:**
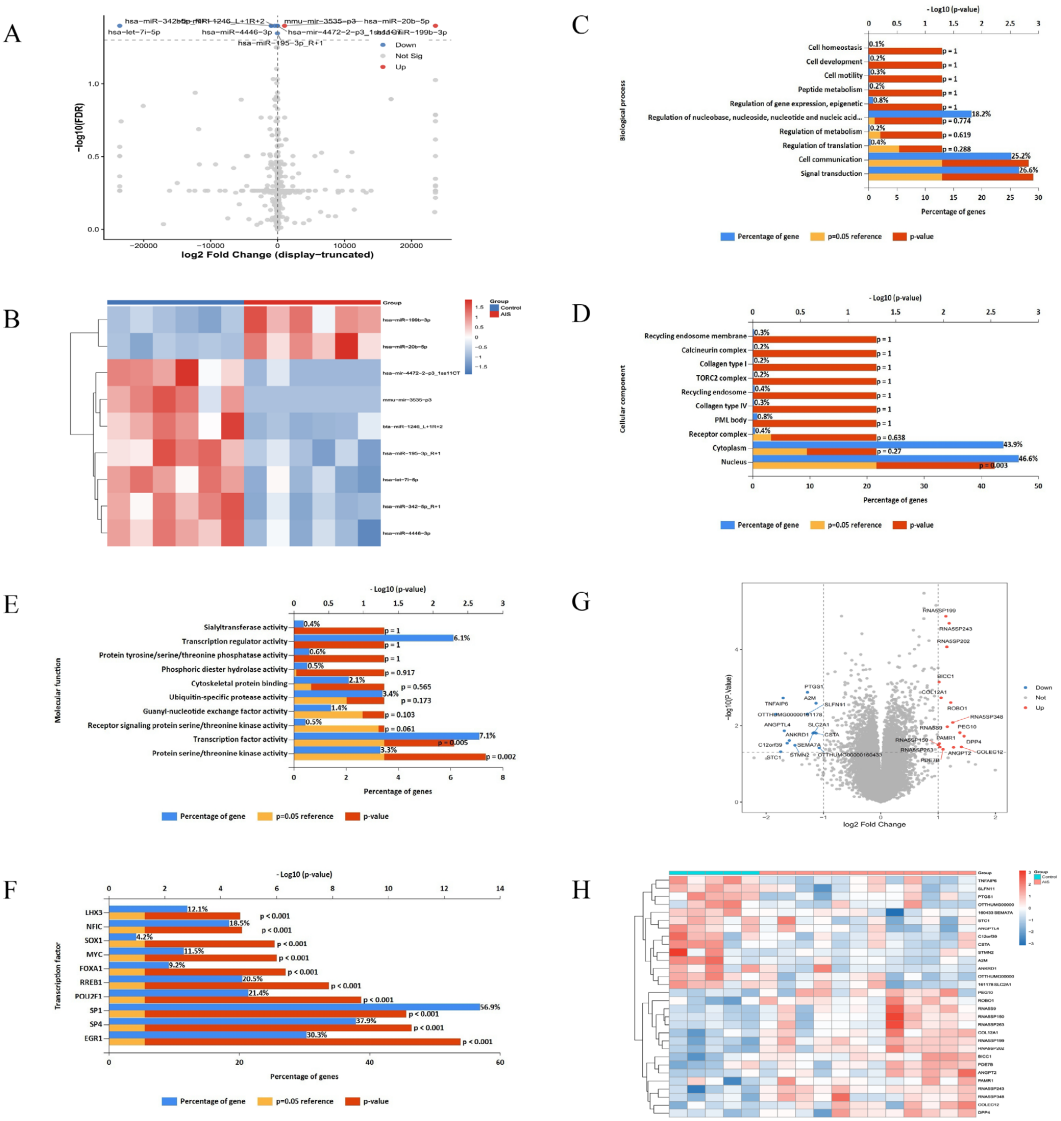
Differential miRNA and mRNA expression profiles in AIS and FunRich‐based functional enrichment analysis of AIS‐associated differentially expressed miRNAs. (A) Volcano plot of differentially expressed miRNAs in GSE235203 identified using |log_2_FC| > 2 and FDR < 0.05. Red and blue dots represent upregulated and downregulated miRNAs, respectively, whereas gray dots represent miRNAs that did not meet the selection threshold. (B) Heatmap of differentially expressed miRNAs in GSE235203. Rows represent miRNAs and columns represent samples; colors indicate row‐wise standardized relative expression levels. (C) Biological Process (BP) enrichment analysis of differentially expressed miRNAs. (D) Cellular Component (CC) enrichment analysis of differentially expressed miRNAs. (E) Molecular Function (MF) enrichment analysis of differentially expressed miRNAs. (F) Transcription factor (TF) enrichment analysis of differentially expressed miRNAs. Blue bars indicate the proportion of genes in each term, and the red line indicates enrichment significance. (G) Volcano plot of differentially expressed mRNAs in the GSE110359 dataset identified using |log_2_FC| > 1 and nominal *p* < 0.05. Red and blue dots represent upregulated and downregulated genes, respectively, whereas gray dots represent genes that did not meet the selection threshold. (H) Heatmap of the 30 differentially expressed mRNAs in GSE110359. Rows represent genes and columns represent samples; colors indicate row‐wise standardized relative expression levels.

A total of nine differentially expressed miRNAs were identified in the plasma miRNA dataset using |log_2_FC| > 2 and FDR < 0.05, including two upregulated and seven downregulated miRNAs (Figure 2A,B). The volcano plot and heatmap showed a distinct miRNA expression pattern between AIS and control samples. FunRich analysis indicated that these differentially expressed miRNAs were mainly enriched in signal transduction, cell communication, and transcriptional regulation (Figure 2C–F). Transcription factor enrichment suggested SP1, SP4, and EGR1 as potential upstream regulatory nodes. Subsequent target prediction yielded 1353 candidate target genes for downstream integrative analysis (Data S1).

In the bone marrow transcriptomic dataset, 30 mRNAs were identified using |log_2_FC| > 1 and nominal *p* < 0.05, including 16 upregulated and 14 downregulated genes (Figure 2G,H). Because of the limited sample size, these mRNA results were interpreted as exploratory transcriptional abnormalities that provided the basis for overlap analysis and downstream biological prioritization rather than as a definitive differential‐expression signature (Data S2).

### 3.2 Cross‐Layer Integration and HERB Mapping Provide a VE‐Related Prioritization Signal

**FIGURE 3:**
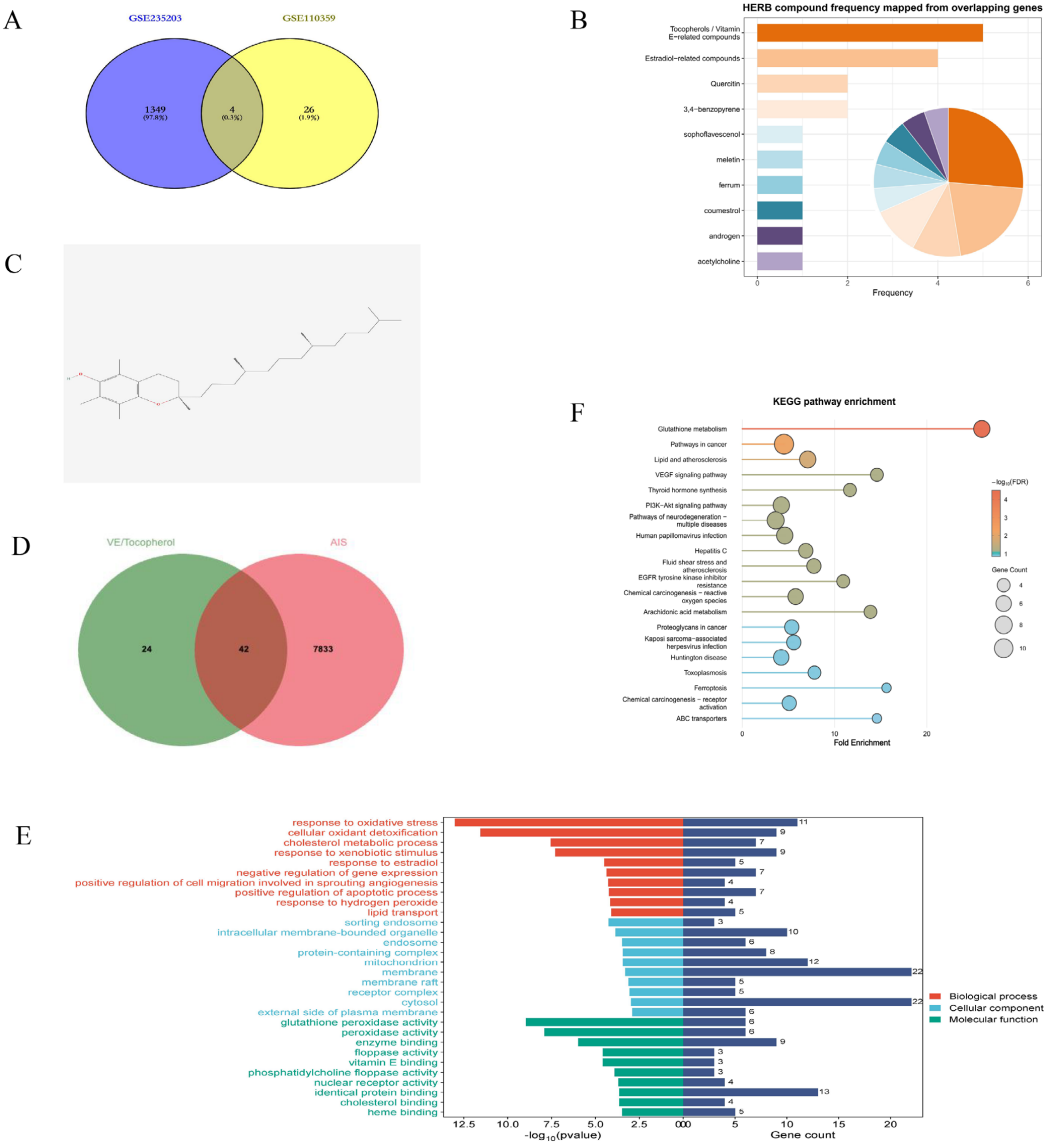
Screening of overlapping targets identifies a vitamin E‐related molecular network and its functional characteristics in AIS. (A) Venn diagram of predicted targets of differentially expressed miRNAs and differentially expressed mRNAs from GSE110359. (B) Composition plot of compounds corresponding to the four overlapping genes identified by HERB 2.0 mapping; this step is interpreted as compound prioritization rather than exposure‐based validation. (C) Molecular formula of vitamin E/tocopherol. (D) Venn diagram of VE/tocopherol‐related targets and AIS‐associated disease targets, showing 42 shared targets. (E) GO enrichment analysis of the shared targets, including Biological Process (BP), Cellular Component (CC), and Molecular Function (MF). (F) KEGG pathway enrichment analysis of the shared targets. Bar length indicates the number of enriched genes, and color intensity represents enrichment significance. Nominal and adjusted statistics are provided in Data S3.

Intersection of 1353 predicted miRNA target genes with 30 differentially expressed mRNAs from GSE110359 yielded four overlapping candidate genes: BICC1, PEG10, PDE7B, and STC1 (Figure 3A). HERB 2.0 compound annotation (Table 2) showed that VE/tocopherol‐related compounds appeared as the most recurrent natural‐ingredient category, followed by estradiol‐related compounds (Figure 3B,C). Notably, STC1 was linked to several tocopherol‐related entries. Because this four‐gene HERB mapping is only a compound‐prioritization signal, it was interpreted as a VE‐associated molecular entry point rather than direct evidence of VE involvement in AIS. Subsequent analyses examined whether a broader VE‐AIS shared‐target network showed biologically coherent enrichment patterns (Data S3).

**TABLE 2:** Candidate compounds corresponding to the four overlapping targets identified by HERB mapping.

Target	Compound	HERB ID
BICC1	3,4‐benzopyrene	HBIN007325
PEG10	17β‐estradiol	HBIN001991
	17β‐oestradiol	HBIN001999
	3,4‐benzopyrene	HBIN007325
	Acetylcholine	HBIN014467
	Androgen	HBIN016005
	Meletin	HBIN034660
	Quercetin	HBIN041721
PDE7B	Sophoflavescenol	HBIN044345
STC1	17β‐estradiol	HBIN001991
	17β‐oestradiol	HBIN001999
	α‐tocopherol	HBIN015727
	Coumestrol	HBIN021620
	Ferrum	HBIN026460
	Quercetin	HBIN041721
	Tocophero^a^	HBIN046505
	Tocopherol	HBIN046506
	Vitamin E (alpha)	HBIN048054
	Vitamin E beta	HBIN048058

*Note:* Compound names were standardized for presentation while retaining the original HERB IDs. Entries with similar names but different HERB IDs were preserved as separate database records.

^a^ The term “tocophero” was retained according to the original HERB record.

### 3.3 VE–AIS Shared Targets Are Enriched in Redox and Glutathione‐Related Pathway Contexts

Integration of 66 VE/tocopherol‐related targets with 7875 AIS disease‐associated targets yielded 42 shared targets (Figure 3D; Data S3). GO enrichment showed FDR‐supported enrichment in response to oxidative stress (11 genes, *p* = 9.91e−14, FDR = 9.38e−11), cellular oxidant detoxification (9 genes, *p* = 2.84e−12, FDR = 1.34e−9), glutathione peroxidase activity (6 genes, *p* = 1.11e−9, FDR = 3.06e−7), and vitamin E binding (3 genes, *p* = 2.65e−5, FDR = 1.46e−3). KEGG enrichment supported glutathione metabolism (7 genes, *p* = 1.96e−7, FDR = 3.19e−5) and the lipid‐inflammatory category annotated as “Lipid and atherosclerosis” (7 genes, *p* = 3.44e−4, FDR = 1.87e−2). Arachidonic acid metabolism, VEGF signaling, PI3K‐Akt signaling, and ferroptosis were retained as nominal or contextual pathway signals. Overall, the strongest support was redox‐buffering and glutathione‐related programs, with lipid‐inflammatory signaling and vascular‐response pathways providing additional context.

### 3.4 Complementary Feature‐Selection Approaches Prioritize Five VE‐AIS Candidate Genes

**FIGURE 4:**
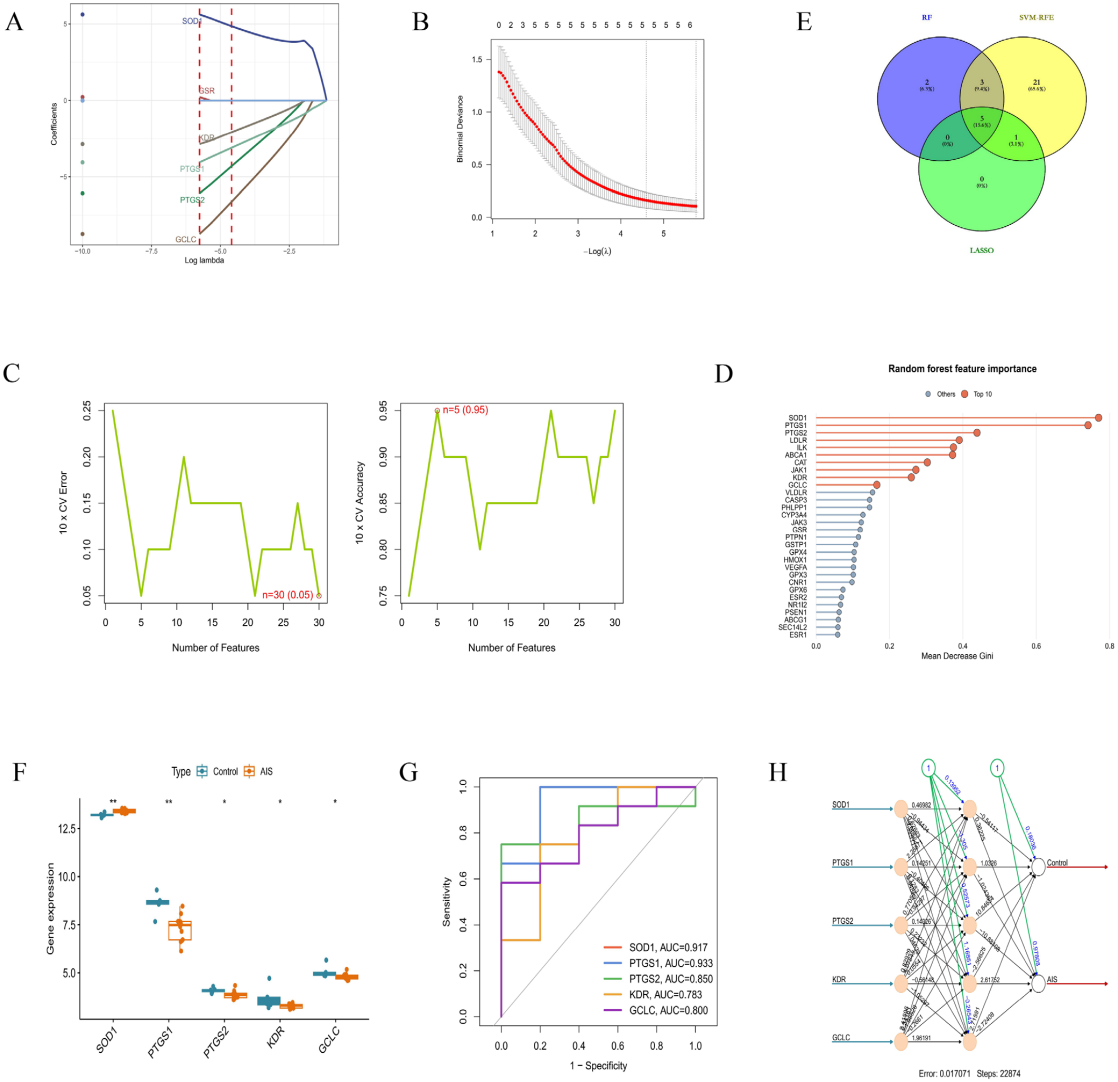
Prioritization and exploratory evaluation of VE–AIS candidate feature genes. (A) Cross‐validation curve of the LASSO logistic regression model. (B) Coefficient profile plot of candidate genes in the LASSO model. (C) Cross‐validation performance of SVM‐RFE. (D) Variable importance ranking derived from the random forest model. (E) Venn diagram showing the overlap among genes identified by LASSO, SVM‐RFE, and random forest, yielding five final key feature genes. (F) Expression differences of the five key feature genes between AIS and control samples. (G) ROC curves of the five key feature genes. (H) Network architecture of the exploratory ANN model based on the prioritized feature genes (shown for descriptive completeness; not interpreted as validated predictive performance).

Based on the shared VE‐AIS targets, LASSO, SVM‐RFE, and random forest were applied to the GSE110359 expression matrix. LASSO retained GCLC, SOD1, GSR, PTGS2, PTGS1, and KDR (Figure 4A,B). SVM‐RFE selected mainly PTGS1, KDR, PTGS2, SOD1, and GCLC (Figure 4C), while random forest ranked SOD1, PTGS1, PTGS2, KDR, and GCLC among the top features (Figure 4D). The cross‐method intersection prioritized SOD1, PTGS1, PTGS2, KDR, and GCLC as candidate molecular nodes for downstream biological interpretation, not as a validated diagnostic feature set (Figure 4E).

Expression analysis showed nominal differences in the five prioritized genes in GSE110359: SOD1 was increased (logFC = 0.200, *p* = 0.00157, adj.*p* = 0.551), whereas PTGS1 (logFC = −1.279, *p* = 0.00133, adj.*p* = 0.517), PTGS2 (logFC = −0.233, *p* = 0.03398, adj.*p* = 0.786), KDR (logFC = −0.396, *p* = 0.03208, adj.*p* = 0.786), and GCLC (logFC = −0.259, *p* = 0.03794, adj.*p* = 0.800) were reduced (Figure 4F; Data S2). Full‐transcriptome adjusted *p* values were not significant, supporting the exploratory interpretation of the feature‐gene set. Single‐gene ROC analysis showed within‐dataset separation patterns, with AUCs of 0.933 for PTGS1, 0.917 for SOD1, 0.850 for PTGS2, 0.800 for GCLC, and 0.783 for KDR (Figure 4G). These ROC results were interpreted only as an internal consistency check. The ANN output is retained in Data S3 for completeness, and was not used to support the main conclusions because the small discovery dataset and lack of independent validation create a substantial risk of overfitting or small‐sample instability.

### 3.5 Feature‐Gene‐Associated Pathways Reflect Redox, Inflammatory, Matrix, and Vascular Remodeling Programs

To determine whether the prioritized VE‐AIS feature genes were associated with distinct functional states within AIS, samples were divided into high‐ and low‐expression groups for each gene, followed by KEGG‐ and GO‐based GSEA analyses. Although the enrichment patterns associated with the five key genes varied, they collectively converged on major functional themes including mitochondrial/proteostasis programs, replication/splicing, inflammation/immunity, adhesion/extracellular matrix remodeling, and vascular/vesicular transport (Figure 6F).

**FIGURE 5:**
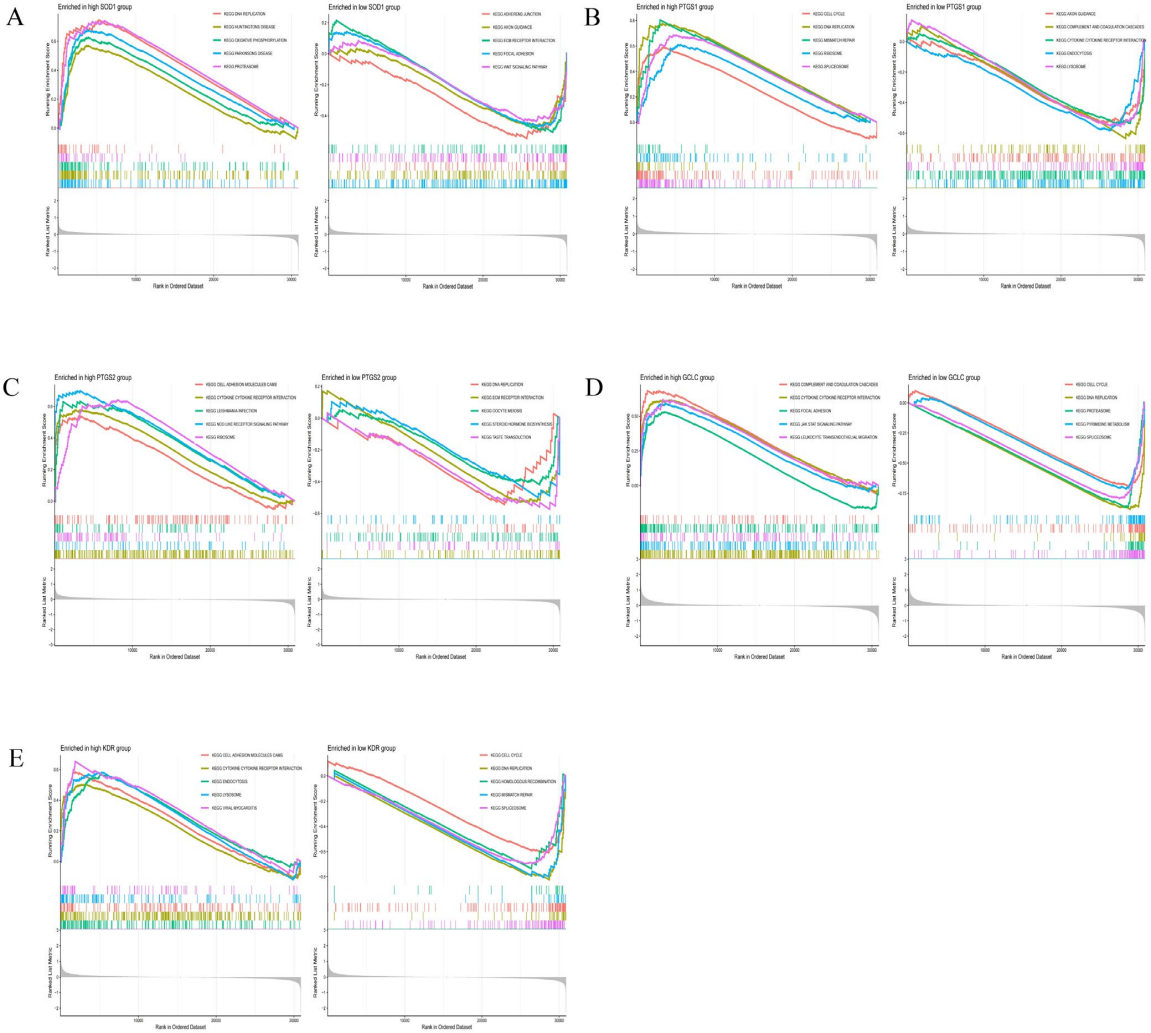
KEGG‐GSEA analysis of key feature genes under different expression states within AIS. (A) Representative KEGG enrichment curves for the high‐ and low‐SOD1 expression groups. (B) Representative KEGG enrichment curves for the high‐ and low‐PTGS1 expression groups. (C) Representative KEGG enrichment curves for the high‐ and low‐PTGS2 expression groups. (D) Representative KEGG enrichment curves for the high‐ and low‐GCLC expression groups. (E) Representative KEGG enrichment curves for the high‐ and low‐KDR expression groups. AIS samples were stratified according to the median expression of each target gene, and representative significantly enriched pathways under high‐ and low‐expression states are shown. Full pathway statistics are provided in Data S4.

**FIGURE 6:**
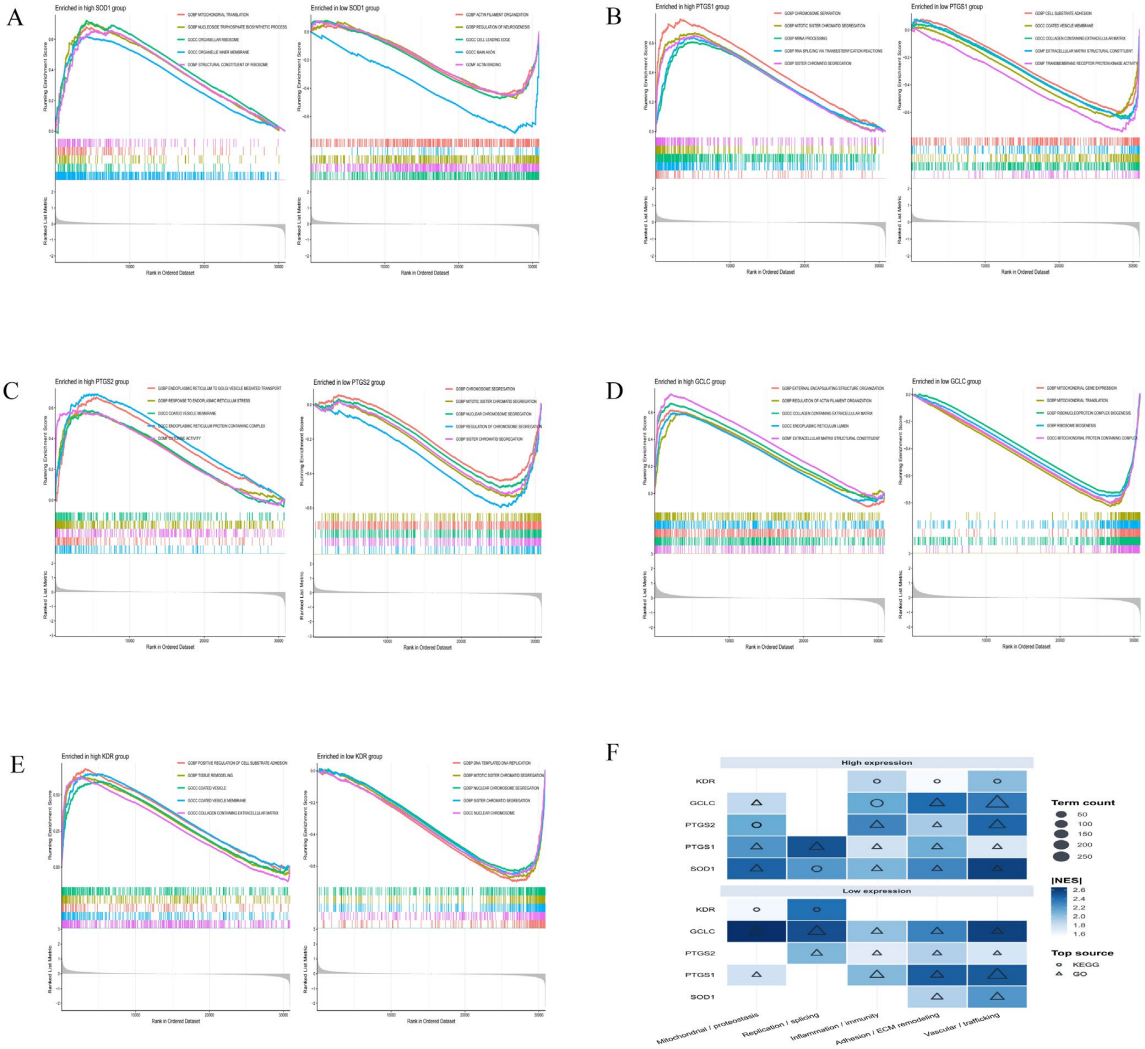
GO‐GSEA analysis of key feature genes under different expression states within AIS. (A) Representative GO enrichment curves for the high‐ and low‐SOD1 expression groups. (B) Representative GO enrichment curves for the high‐ and low‐PTGS1 expression groups. (C) Representative GO enrichment curves for the high‐ and low‐PTGS2 expression groups. (D) Representative GO enrichment curves for the high‐ and low‐GCLC expression groups. (E) Representative GO enrichment curves for the high‐ and low‐KDR expression groups. (F) Functional theme matrix of intradisease pathway stratification in AIS defined by the key feature genes. AIS samples were divided into high‐ and low‐expression groups according to the median expression of SOD1, PTGS1, PTGS2, GCLC, and KDR, followed by KEGG‐GSEA and GO‐GSEA analyses. The figure summarizes significantly enriched signals across functional themes for different genes. Color indicates enrichment intensity, dot size indicates the number of significant terms, and dot shape indicates the primary enrichment source (KEGG or GO). Full results are provided in Data S4.

Gene‐specific enrichment patterns further supported the biological interpretation of the five prioritized genes. High SOD1 expression was mainly associated with oxidative phosphorylation, proteasome function, and DNA replication, whereas low SOD1 expression was linked to adhesion and extracellular‐matrix pathways (Figures 5A and 6A). PTGS2 showed the clearest immune‐inflammatory pattern, with high expression linked to cytokine, chemokine, NOD‐like receptor, and antigen‐presentation pathways (Figures 5C and 6C). KDR‐associated programs were connected to adhesion, cytokine signaling, endocytosis, and tissue remodeling, supporting its relationship to vascular and stromal‐response programs (Figures 5E and 6E). PTGS1 and GCLC provided additional links to cell‐cycle/proteostasis regulation, cytokine signaling, glutathione‐related regulation, and extracellular‐matrix remodeling (Figures 5B,D and 6B,D; Data S4). Together, these findings suggest that the five prioritized genes reflect coordinated redox, inflammatory, remodeling, and vascular‐response programs within AIS, although the GSEA results should be interpreted as exploratory pathway‐context signals.

### 3.6 Single‐Cell Atlas Localization of Key Genes Suggests Region‐Specific Stress‐Vascular Response Programs

**FIGURE 7:**
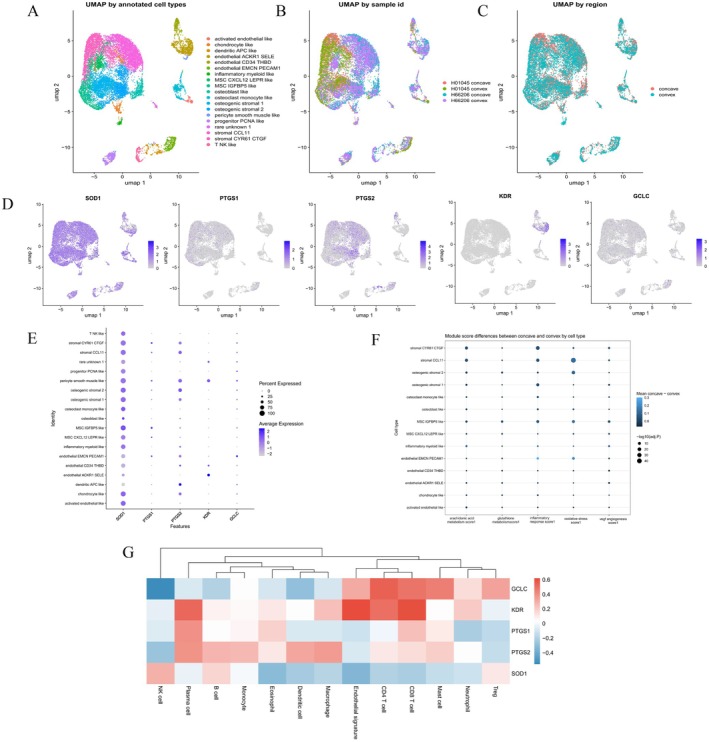
Single‐cell atlas of AIS local tissues suggests the cellular localization of key feature genes and region‐specific stress‐vascular response‐related programs. (A) UMAP clustering of single‐cell transcriptomes from AIS local tissues, colored by annotated cell type. (B) UMAP distribution colored by sample origin. (C) UMAP distribution colored by anatomical region (concave versus convex). (D) FeaturePlot showing the expression distributions of the key feature genes SOD1, PTGS1, PTGS2, KDR, and GCLC across the single‐cell atlas. (E) DotPlot showing the expression patterns of these key feature genes across different cell populations. Dot size indicates the proportion of expressing cells, and color indicates average expression level. (F) Descriptive differences in functional module scores between concave and convex regions across distinct cell populations, involving oxidative stress, glutathione metabolism, arachidonic acid metabolism, VEGF/angiogenesis, and inflammatory‐response programs. (G) Spearman correlation heatmap showing correlations between the key feature genes and immune signatures in the bulk transcriptomic dataset. Concave‐versus‐convex displays are descriptive because cells were nested within two AIS donors.

Single‐cell analysis of AIS paraspinal muscle tissue identified 19 annotated cell populations, including stromal/osteogenic‐related cells, MSC‐like cells, endothelial‐related cells, chondrocyte‐like cells, and immune‐related cell populations (Figure 7A). Global clustering showed broadly shared cell identities across samples, while concave and convex regions displayed descriptive differences in the relative abundance and functional states of selected cell populations (Figure 7B,C). Because the public dataset contains four libraries from only two AIS donors, concave‐convex comparisons were treated as descriptive region‐level contrasts rather than as donor‐independent statistical validation.

FeaturePlot analysis showed that KDR and PTGS2 exhibited relatively localized, cell type‐specific distributions, whereas SOD1 displayed broader expression across cell populations (Figure 7D). Average‐expression summaries supported these localization patterns: PTGS2 was highest in dendritic/APC‐like cells, KDR was highest in endothelial_ACKR1_SELE cells, GCLC was highest in endothelial_EMCN_PECAM1 cells, PTGS1 was highest in MSC_IGFBP5_like cells, and SOD1 was highest in osteoblast‐like cells (Figure 7E; Data S5 and S6).

Module scoring suggested that oxidative stress, glutathione metabolism, arachidonic acid metabolism, VEGF/angiogenesis, and inflammatory‐response programs were mainly concentrated in endothelial‐related cells and a subset of stromal/MSC‐like cells, with descriptive directional differences between concave and convex regions (Figure 7F). Combined with ssGSEA and correlation analysis at the bulk‐transcriptome level, KDR was positively correlated with the endothelial signature, whereas PTGS2 showed a stronger tendency to correlate with myeloid/APC‐like inflammatory signatures (Figure 7G; Data S6).

### 3.7 Cell–Cell Communication Analysis Predicts a Local Inflammatory‐Vascular‐Stromal Remodeling Context in AIS

**FIGURE 8:**
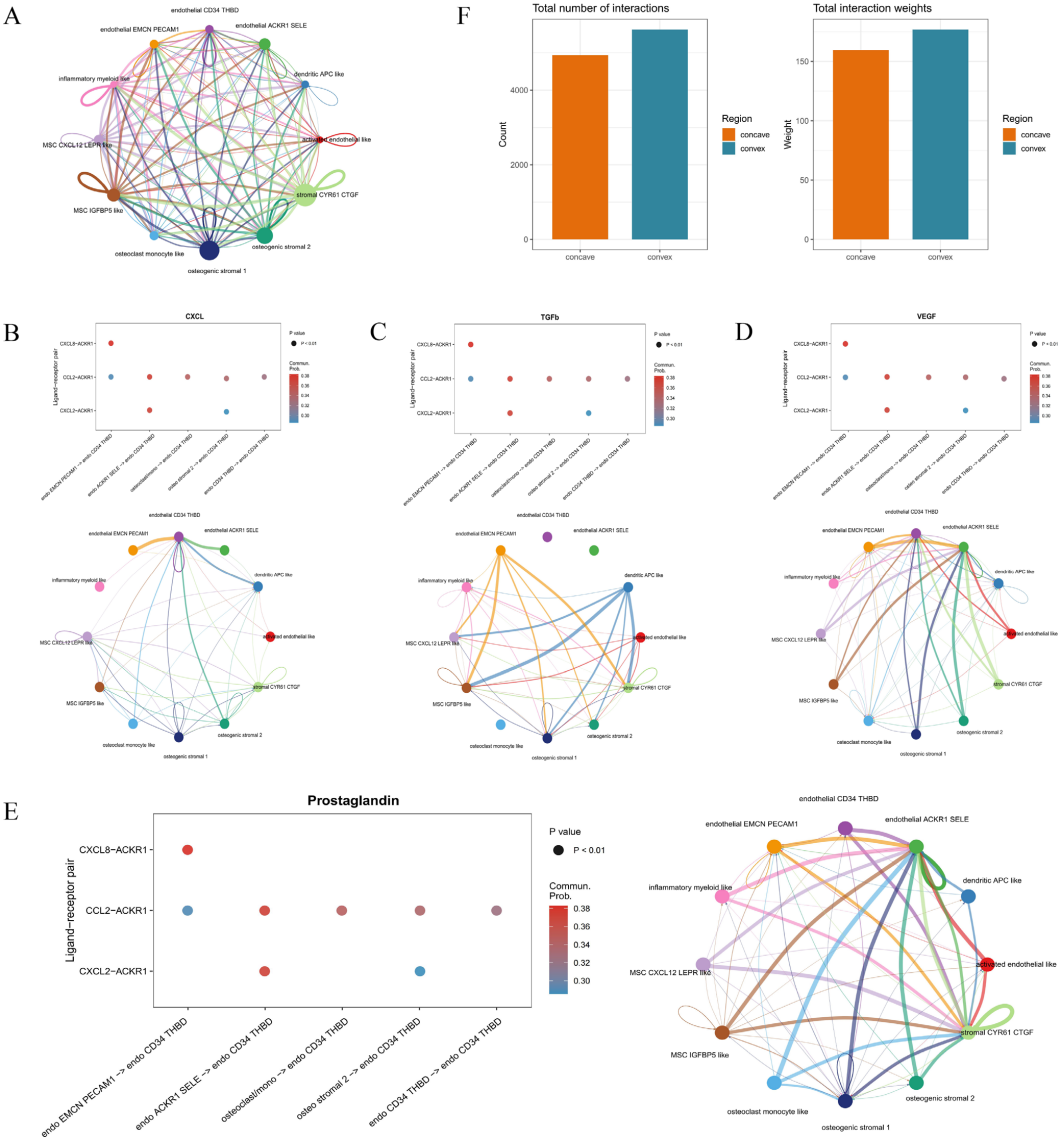
Cell–cell communication analysis suggests a predicted local inflammatory‐vascular‐stromal remodeling network in AIS. (A) Global communication network among key cell populations. This network is shown as a predicted communication context rather than as direct experimental validation. (B–E) Bubble plots and network plots of representative ligand–receptor signaling pathways, showing the activity of (B) VEGF, (C) CXCL, (D) TGFβ, and (E) prostaglandin signaling across different cell combinations. (F) Descriptive comparison of predicted overall communication strength between concave and convex regions. Because the public single‐cell dataset includes a limited number of donors, this panel is descriptive rather than donor‐independent statistical validation.

CellChat analysis of the selected key cell populations predicted 3986 focused sender‐receiver interactions across 96 detected signaling pathways within the AIS local microenvironment (Data S7). These predicted interactions primarily involved inflammatory/APC‐like cells, endothelial cells, MSC‐like cells, and osteogenic stromal cells (Figure 8A). The overall communication network suggested that multiple cell types may engage in ligand‐receptor interactions, consistent with a multicellular remodeling context in AIS, but these outputs should be interpreted as predicted communication patterns rather than validated signaling activity.

Further pathway‐level analysis predicted signaling pathways such as VEGF, CXCL, TGFβ, and Prostaglandin across multiple cell combinations. Representative interactions included activated_endothelial_like to dendritic/APC‐like VEGFA‐FLT1/VEGFR1 signaling, endothelial_ACKR1_SELE to endothelial_CD34_THBD CXCL1‐ACKR1 signaling, dendritic/APC‐like to inflammatory myeloid‐like TGFB1‐TGFBR1/TGFBR2 signaling, and dendritic/APC‐like to stromal_CYR61_CTGF prostaglandin signaling (Figure 8B–E; Data S7). Descriptive comparison between regions showed that both the total interaction count and interaction weight were numerically higher in the concave region than in the convex region, suggesting predicted stronger intercellular communication on the concave side (Figure 8F).

### 3.8 Structural Analyses Provide Preliminary Computational Context for α‐Tocopherol and Selected VE‐AIS Candidate Proteins

Molecular docking assessed preliminary computational structural compatibility between α‐tocopherol and structurally analyzed VE‐AIS candidate proteins, including KDR, PTGS1, PTGS2, and SOD1. GCLC was excluded from structural interpretation because MD output was unavailable. α‐Tocopherol showed favorable predicted docking scores of −5.3 kcal/mol for KDR, −7.0 kcal/mol for PTGS1, −7.7 kcal/mol for PTGS2, and −6.2 kcal/mol for SOD1. Among these, PTGS2 displayed the lowest docking energy with the current computational setup, but these values are predicted energetic estimates within the selected search grids, not experimental evidence of binding affinity or functional regulation.

**FIGURE 9:**
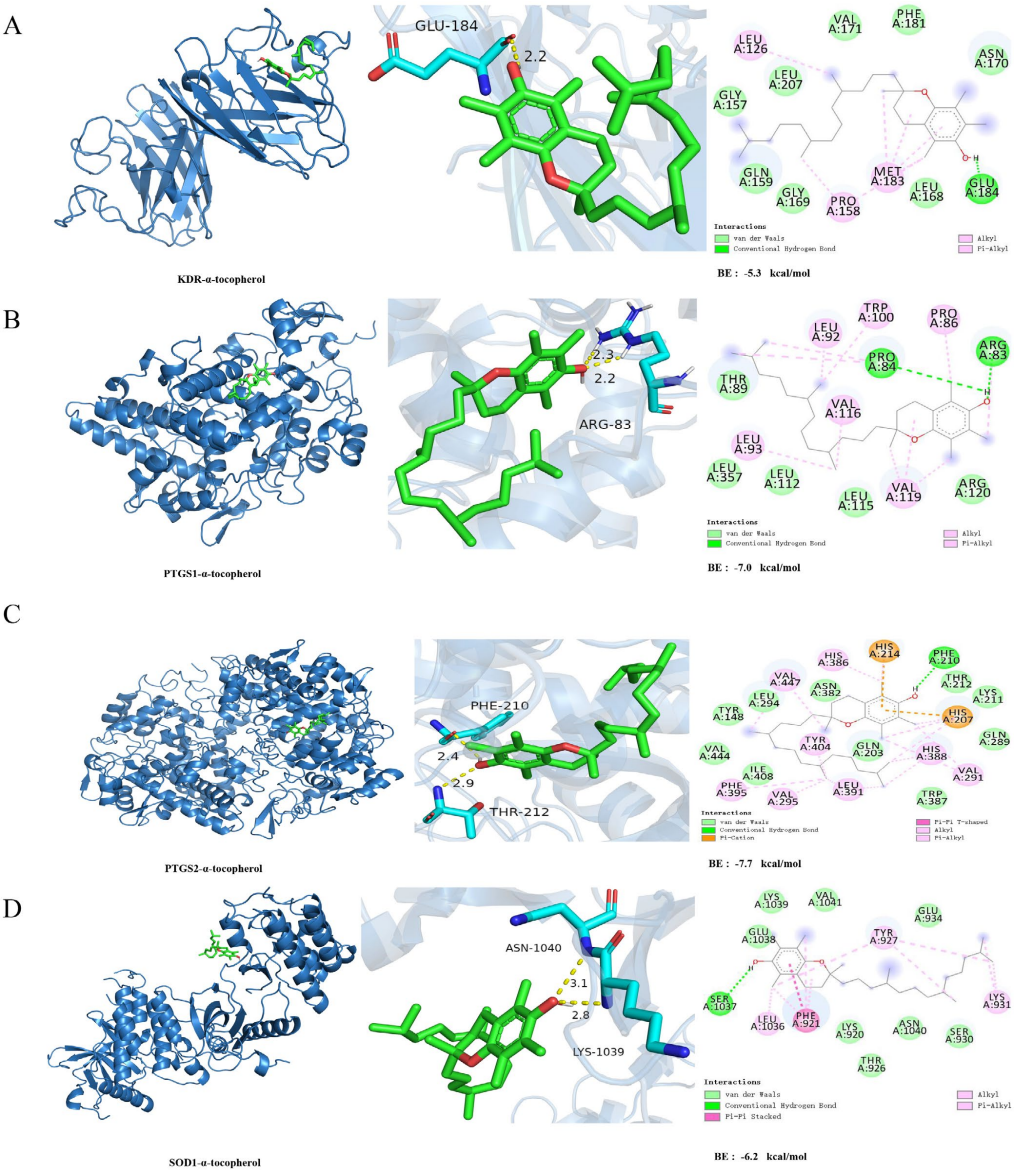
Molecular docking analysis of α‐tocopherol with key AIS target proteins. (A) Three‐dimensional binding mode and two‐dimensional interaction diagram of the KDR–α‐tocopherol complex. (B) Three‐dimensional binding mode and two‐dimensional interaction diagram of the PTGS1–α‐tocopherol complex. (C) Three‐dimensional binding mode and two‐dimensional interaction diagram of the PTGS2–α‐tocopherol complex. (D) Three‐dimensional binding mode and two‐dimensional interaction diagram of the SOD1–α‐tocopherol complex. The figure shows the spatial positioning of α‐tocopherol within the binding pockets of different target proteins and its major interactions with key amino acid residues, including hydrogen bonds, hydrophobic interactions, and π‐related interactions. These docking results are interpreted as structural plausibility rather than proof of functional binding in AIS.

In the predicted docking poses, within the KDR–α‐tocopherol complex, α‐tocopherol formed a hydrogen bond with GLU184 and hydrophobic interactions with LEU126, MET183, and PRO158 (Figure 9A). In the PTGS1–α‐tocopherol complex, the ligand formed hydrogen bonds with ARG83 and PRO84, along with major hydrophobic interactions involving VAL119, TRP100, and PRO86 (Figure 9B). In the PTGS2–α‐tocopherol complex, α‐tocopherol formed a hydrogen bond with PHE210, hydrophobic interactions with HIS386, PHE395, LEU391, and TYR404, and π‐cation interactions with HIS214 and HIS207 (Figure 9C). In the SOD1–α‐tocopherol complex, the ligand formed a hydrogen bond with SER1037 and hydrophobic interactions with TYR927, PHE921, and LEU1036, with PHE921 additionally participating in a π–π stacking interaction (Figure 9D). These interaction patterns describe predicted docking poses only and should not be interpreted as experimentally validated binding modes.

**FIGURE 10:**
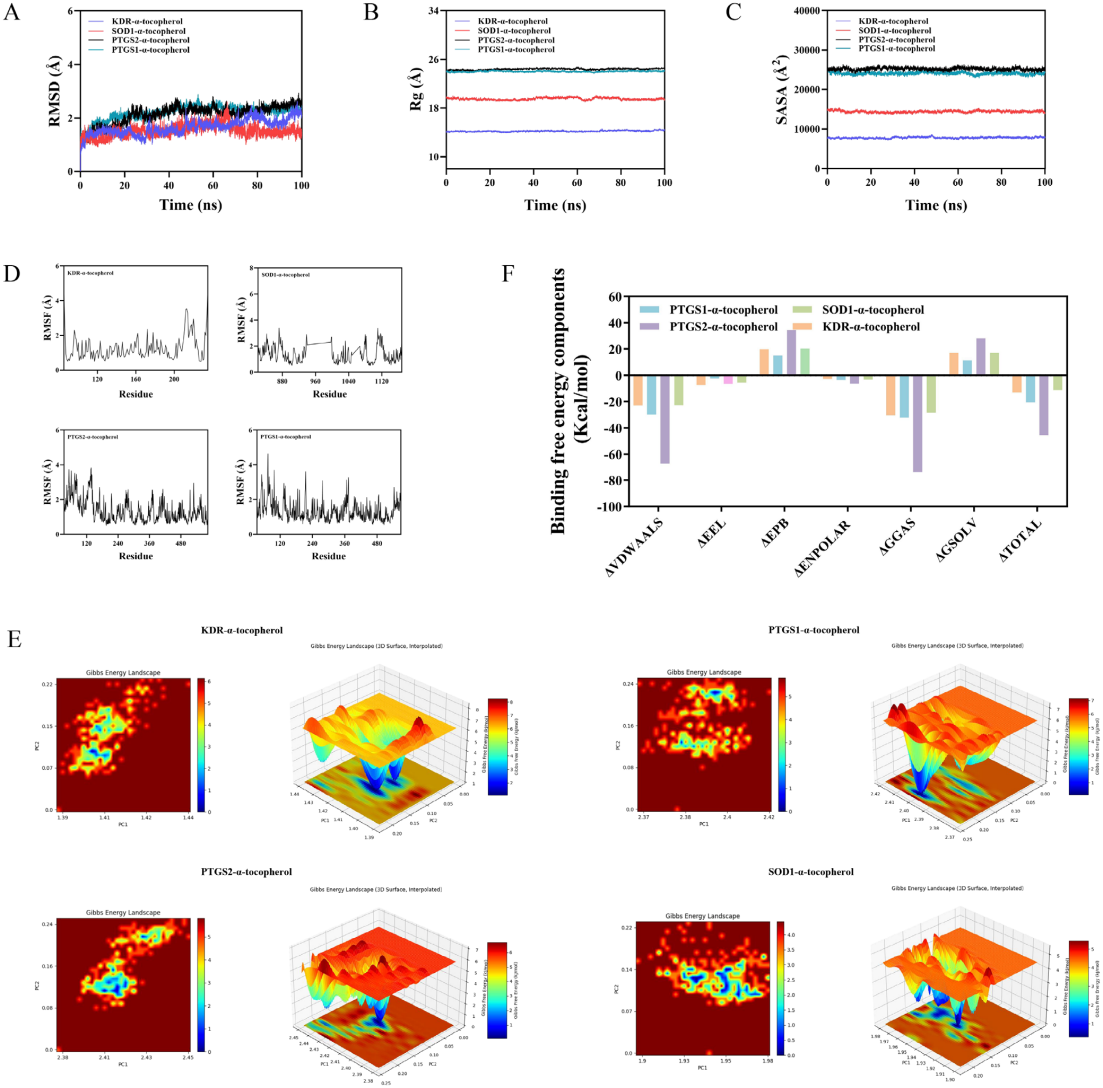
Molecular dynamics simulation analysis of α‐tocopherol–target protein complexes. (A) RMSD trajectories of the four protein–ligand complexes over the 100 ns simulation. (B) Radius of gyration (Rg) trajectories of the four complexes. (C) Solvent‐accessible surface area (SASA) trajectories of the four complexes. (D) RMSF profiles of the four complexes. (E) Free energy landscape (FEL) plots of the four complexes. These results were used to evaluate the conformational stability and energy distribution characteristics of α‐tocopherol in complex with different target proteins during dynamic simulation. The MD outputs are interpreted as computational structural context rather than experimental confirmation of target regulation. (F) Total binding free energies of the KDR–α‐tocopherol, PTGS1–α‐tocopherol, PTGS2–α‐tocopherol, and SOD1–α‐tocopherol complexes. More negative values indicate stronger predicted interaction tendency within this computational framework; PTGS2 showed the most favorable MM/PBSA estimate.

Subsequent 100 ns MD simulations suggested that the SOD1‐α‐tocopherol complex reached a relatively stable state after approximately 10 ns, whereas the PTGS2‐ and PTGS1–α‐tocopherol complexes showed lower RMSD fluctuation after approximately 40 ns. In contrast, the KDR–α‐tocopherol complex exhibited a continuously rising RMSD trend throughout the simulation (Figure 10A), suggesting lower trajectory stability under the current simulation settings. The Rg and SASA values of all complexes showed only minor overall fluctuations, without obvious abnormal changes (Figure 10B,C). RMSF analysis further indicated relatively limited residue‐level fluctuation amplitudes across most regions of the modeled complexes (Figure 10D). Free energy landscape analysis showed that the four complexes occupied relatively well‐defined low‐energy conformational regions (Figure 10E).

MM/PBSA analysis estimated interaction free energies of −13.12, −20.61, −45.44, and −11.14 kcal/mol for the KDR, PTGS1, PTGS2, and SOD1 complexes, respectively (Figure 10F). Among these, the PTGS2 complex showed the most favorable MM/PBSA estimate within the current computational framework. These structural analyses therefore provide computational plausibility for protein‐level compatibility, but they do not establish biological target engagement, nutritional relevance, or functional modulation in AIS tissues.

### 3.9 NHANES Analysis Provides External Adolescent Clinical Nutrition Context

The NHANES adolescent analysis included 1582 participants aged 10–19 years before endpoint‐specific missingness filtering. The weighted mean serum α‐tocopherol concentration was 20.11 μmol/L, and the weighted mean α‐tocopherol/total cholesterol ratio was 5.00 μmol/mmol. The weighted mean dietary α‐tocopherol intake was 7.94 mg/day, and the weighted mean lumbar spine BMD was 0.929 g/cm^2^ (Data S10).

In weighted multivariable models, neither serum α‐tocopherol/total cholesterol nor dietary vitamin E density was associated with lumbar spine BMD after adjustment. By contrast, serum 25‐hydroxyvitamin D and BMI were positively associated with lumbar spine BMD in the same model, supporting the ability of the NHANES dataset to detect established bone‐related associations (Table 3). Serum α‐tocopherol/total cholesterol was nominally inversely associated with BMI, whereas serum γ‐tocopherol/total cholesterol was nominally positively associated with BMI. Serum γ‐tocopherol/total cholesterol, but not α‐tocopherol/total cholesterol, was nominally associated with higher log‐transformed hs‐CRP levels. Thus, NHANES contributed population‐level adolescent nutritional context, rather than AIS‐specific validation, and mainly suggested links between lipid‐adjusted tocopherol measures and broader metabolic/inflammatory variables (Data S10).

**TABLE 3:** Exploratory NHANES associations of vitamin E exposure/status with adolescent skeletal, metabolic, inflammatory, and pubertal variables.

Exposure (unit)	Outcome	*N*	β	95% CI	*p*
α‐toc/chol (μmol/mmol)	Lumbar spine BMD (g/cm^2^)	1010	−0.0053	−0.0141 to 0.0035	0.241
Dietary VE density (mg/1000 kcal)	Lumbar spine BMD (g/cm^2^)	870	−0.0074	−0.0190 to 0.0043	0.216
α‐toc/chol (μmol/mmol)	BMI (kg/m^2^)	1208	−0.779	−1.280 to −0.278	0.002
γ‐toc/chol (μmol/mmol)	BMI (kg/m^2^)	1208	1.431	1.019 to 1.842	< 0.001
γ‐toc/chol (μmol/mmol)	Log‐transformed hs‐CRP	1198	0.108	0.0329 to 0.183	0.005
α‐toc/chol (μmol/mmol)	Age at menarche (years)	421	−0.104	−0.285 to 0.0762	0.257
Post‐menarche status	α‐toc/chol (μmol/mmol)	452	−0.105	−0.720 to 0.509	0.737

*Note:* NHANES analyses are exploratory external adolescent nutrition context and are not AIS‐specific validation. *N* represents the unweighted analytic sample size for each model. β estimates were obtained from weighted regression models accounting for NHANES sampling weights, strata, and primary sampling units. Serum biomarker models used NHANES examination weights, whereas dietary models used 2‐day dietary weights. Continuous vitamin E exposure/status variables were z‐standardized; therefore, β represents the change in outcome per 1 SD increase in the exposure variable, unless otherwise specified.

Abbreviations: BMD, bone mineral density; hs‐CRP, high‐sensitivity C‐reactive protein; toc/chol, tocopherol/total cholesterol; VE, vitamin E.

Dietary vitamin E density was not associated with BMI or hs‐CRP after adjustment. However, energy‐adjusted dietary vitamin E intake was positively associated with serum α‐tocopherol, but not serum γ‐tocopherol, supporting partial consistency between dietary vitamin E exposure and circulating α‐tocopherol status.

Among female participants aged 12–19 years, serum α‐tocopherol/total cholesterol was not associated with age at menarche. Post‐menarche status was also not associated with serum α‐tocopherol/total cholesterol after adjustment for age, race/ethnicity, BMI, and serum 25‐hydroxyvitamin D. Fasting lipid‐adjustment sensitivity analyses similarly showed no association between lipid‐adjusted α‐tocopherol and lumbar spine BMD or log‐transformed hs‐CRP (Data S10).

Given the multiple exposure and endpoint comparisons, these NHANES associations were interpreted as nominal adolescent nutrition context and not as corrected evidence for vitamin E‐related skeletal, inflammatory, pubertal, or AIS‐specific effects.

### 3.10 Supplementary AIS Progression‐Related miRNA Contextual Mapping Using GSE286204

A supplementary exploratory analysis of GSE286204 showed that published AIS progression‐associated circulating miRNA signatures provided descriptive molecular context for extracellular‐matrix/osteogenic, inflammatory, lipid‐inflammatory, and vascular‐remodeling modules. However, GSE286204 did not include dietary vitamin E intake, serum tocopherol measurements, lipid‐adjusted tocopherol variables, or oxidative‐stress biomarkers. Therefore, this analysis was interpreted only as AIS progression‐related molecular context and not as validation of a vitamin E–Cobb angle or vitamin E–progression relationship.

## 4 Discussion

This study develops a hypothesis‐generating molecular framework in which database‐derived VE‐related signatures overlap with AIS‐associated redox, lipid‐inflammatory, and vascular‐response programs. By narrowing 42 VE‐AIS shared targets to five prioritized feature genes (SOD1, GCLC, PTGS1, PTGS2, and KDR), the analysis resolved a broad nutrient‐associated network into three interpretable components: redox buffering, lipid‐inflammatory signaling, and vascular response. These components provide a framework for interpreting multicellular tissue‐remodeling hypotheses in AIS. Rather than supporting a direct effect of vitamin E intake, tocopherol status, or supplementation on AIS, the current results should be interpreted as identifying testable molecular hypotheses for future nutritional, biochemical, and cellular validation.

These findings are consistent with the view that AIS involves both systemic and local tissue dysregulation. Low bone mass, impaired osteogenic differentiation, altered bone marrow signaling, and circulating miRNA abnormalities suggest that AIS‐related molecular changes extend across several biological compartments (Liang et al. 2025; Zhang et al. 2023). In this study, plasma miRNA and bone marrow mRNA profiles were used as complementary analytical entry points to identify convergent molecular abnormalities, rather than to infer direct inter‐tissue causal relationships. This distinction is important because the present findings should be interpreted as cross‐level biological convergence rather than evidence that circulating, bone marrow, and local paraspinal changes occur in a linear sequence.

One central finding is that the four‐gene HERB compound annotation and the broader VE‐AIS shared‐target enrichment converged on redox homeostasis, lipid metabolism, and vascular biology. The HERB result should be viewed as a prioritization signal for selecting a VE‐related molecular context, not as causal evidence that VE exposure is involved in AIS. Although VE is often viewed primarily as a lipid‐soluble antioxidant, tocopherols and their metabolites also regulate COX/eicosanoid metabolism, inflammatory transcriptional programs, and vascular‐response signaling (Jiang 2022; Reiter et al. 2007). This broader biochemical role helps explain why FDR‐supported redox/glutathione terms and lipid‐metabolic KEGG signals were observed, while arachidonic acid metabolism, VEGF signaling, PI3K‐Akt signaling, and ferroptosis were retained as contextual pathway signals. Taken together, these patterns are more consistent with coordinated pathway prioritization than with a single isolated molecular effect.

PTGS1 and PTGS2 provide a biologically interpretable enzymatic link between the tocopherol‐mapped network and prostaglandin‐mediated lipid‐inflammatory signaling. Although the PTGS2/COX‐2 pathway is often regarded as pro‐inflammatory, its biological effects are highly tissue‐, stage‐, and context‐dependent (Martín‐Vázquez et al. 2023; Ricciotti and FitzGerald 2011). In the present analysis, PTGS1 and PTGS2 were reduced in bone marrow‐derived AIS samples, whereas PTGS2‐associated functional programs were linked to cytokine, chemokine, NOD‐like receptor, and antigen‐presentation pathways. At the single‐cell level, PTGS2 was predominantly localized to dendritic/APC‐like cells, and CellChat analysis suggested that prostaglandin signaling was embedded within the inflammatory–stromal communication context of AIS. These findings prioritize PTGS2 as a compartment‐sensitive lipid‐inflammatory candidate node in AIS, rather than a simple marker of increased or decreased inflammation. Structural analyses provided computational context for PTGS2 because α‐tocopherol showed the most favorable predicted compatibility with PTGS2 among the tested proteins, although this remains computational rather than functional evidence. Beyond this lipid‐inflammatory axis, the pathway landscape also pointed to vascular‐response programs that may operate in parallel and potentially interact with inflammatory remodeling within the AIS microenvironment.

KDR represents the vascular‐response component of the proposed VE‐associated axis. KDR encodes VEGFR2, a major receptor mediating VEGF‐induced endothelial responses, angiogenesis, endothelial homeostasis, and vascular repair in remodeling tissue microenvironments (Lee et al. 2007; Santos et al. 2007; Simons et al. 2016; Zhong and Yu 2023). Although KDR was reduced in bone marrow‐derived AIS samples, it was localized mainly to endothelial‐related cells in the single‐cell atlas and correlated positively with endothelial signatures. CellChat analysis further suggested that VEGF signaling was concentrated in endothelial‐related interactions within the local AIS microenvironment, while overall interaction count and communication weight were numerically higher in the concave region than in the convex region. These findings are consistent with a regionally stronger predicted endothelial‐response program integrated with inflammatory and stromal compartments, but do not establish whether angiogenic signaling is increased, decreased, compensatory, or secondary to local remodeling.

SOD1 and GCLC define the redox‐buffering component of the network. In bone marrow‐derived AIS samples, increased SOD1 may reflect compensatory antioxidant activity (Xing et al. 2002), while reduced GCLC may suggest weakened glutathione‐related buffering capacity (Lapenna 2023). Biologically, SOD1 contributes to superoxide detoxification and broader stress‐response regulation, whereas GCLC is the rate‐limiting enzyme for glutathione synthesis and is closely linked to resistance against lipid peroxidation and ferroptosis (Chen et al. 2025; Franklin et al. 2009; Gomez and Germain 2019; Lapenna 2023; Lu 2009; Xu et al. 2022). SOD1 was nominally increased and GCLC was nominally reduced in bone marrow‐derived AIS samples, suggesting a possible imbalance between superoxide‐detoxifying responses and glutathione‐dependent antioxidant capacity. This interpretation is consistent with the redox/glutathione enrichment results and their localization to endothelial and MSC/stromal‐like compartments in the single‐cell analysis. Because glutathione availability supports GPX4‐mediated protection against lipid peroxidation and ferroptosis (Chen et al. 2025; Lu 2009), the SOD1–GCLC module provides a biologically coherent link between antioxidant buffering and lipid oxidative stress, and AIS‐relevant tissue remodeling. However, these transcriptomic and enrichment‐based findings do not directly demonstrate oxidative damage, antioxidant capacity, glutathione depletion, or ferroptosis activity; thus, biochemical and functional validation will be required.

A further contribution of this study is the integration of circulating, bone marrow, and local single‐cell data into a unified interpretive framework. Previous AIS studies have often examined circulating molecular changes, bone metabolic abnormalities, or paraspinal tissue asymmetry as largely separate observations (Chen et al. 2022; Park et al. 2009; Raimondi et al. 2026; Shao et al. 2023). By combining these layers, the present analysis suggests that AIS‐associated abnormalities may converge on a multicellular network involving immune/APC‐like, endothelial, and stromal/osteogenic‐related cells. Because the datasets represent different cohorts and biological compartments, the integrated analysis should be viewed as evidence of parallel biological signals across layers rather than as proof of direct molecular continuity or causal transmission.

Population‐level NHANES analyses further contextualized the molecular findings within adolescent clinical nutrition. However, the results did not support a simple model in which higher vitamin E exposure or α‐tocopherol status was associated with higher lumbar spine BMD or more advanced pubertal maturation. Instead, lipid‐adjusted tocopherol measures were more clearly related to BMI and hs‐CRP, particularly for γ‐tocopherol, suggesting that circulating tocopherol isoforms may reflect broader adiposity‐, lipid‐metabolic‐, and inflammation‐related context. Because these analyses were exploratory and NHANES lacks AIS diagnosis and disease‐severity variables, these findings should be interpreted as external nutritional context rather than AIS‐specific validation.

The supplementary GSE286204 analysis provided AIS progression‐related molecular context by showing descriptive overlap between published progressor‐associated miRNA targets and the redox/glutathione, prostaglandin‐related, vascular‐response, and inflammatory modules. Because GSE286204 lacks vitamin E exposure or serum tocopherol data and the overlap/correlation analyses were not FDR‐significant, this component was not interpreted as validation of a VE–Cobb angle or VE–progression relationship.

From a food and nutrition perspective, VE is a family of diet‐derived, lipid‐soluble tocopherols and tocotrienols whose intake, absorption, transport, and circulating concentrations are shaped by dietary fat intake and lipid metabolism. Beyond antioxidant activity, these compounds may protect membranes, modulate lipid peroxidation, regulate inflammatory mediators, and support endothelial function (Ungurianu et al. 2021). This distinction is important because dietary VE exposure and serum tocopherol status are related but not interchangeable. The present findings should not be construed as evidence that VE intake, serum tocopherol levels, or VE supplementation influence AIS risk, severity, or progression, and they do not justify nutritional or clinical recommendations. Instead, they identify VE‐associated redox, glutathione‐related, lipid‐inflammatory, and vascular‐response pathways that may provide molecular context for future AIS studies incorporating dietary assessment, lipid‐adjusted serum tocopherols, and clinically anchored skeletal or inflammatory outcomes.

Several limitations should be acknowledged. First, the peripheral blood, bone marrow, and paraspinal muscle datasets were derived from different cohorts and tissue compartments; therefore, the findings cannot establish causal transmission among circulating, bone marrow, and local alterations. Second, although NHANES included measured dietary and serum tocopherol variables, it was not AIS‐specific and cannot validate vitamin E–AIS associations. Third, HERB‐based compound mapping and VE‐AIS shared‐target analysis are hypothesis‐generating and cannot establish causality for VE; the four‐gene HERB result should be considered only a prioritization signal. Statistical robustness also varied across analytical layers: FDR‐supported enrichment results provide stronger support for redox/glutathione‐related pathways, whereas nominal‐only differential‐expression, GSEA, NHANES, and progression‐context findings should be interpreted as exploratory signals requiring independent validation. Fourth, the feature‐selection workflow, particularly the ANN output, requires resampling‐based stability assessment and independent validation before any predictive interpretation. The apparent ANN AUC of 1.000 should be interpreted cautiously because it may reflect overfitting or small‐sample instability rather than generalizable diagnostic performance. Fifth, the single‐cell analysis used a limited public dataset from two AIS donors, so concave–convex and CellChat outputs should be interpreted as descriptive or predicted patterns rather than donor‐independent evidence. Sixth, molecular docking and MD simulations provide structural plausibility only and cannot substitute for biochemical binding assays or functional validation. Finally, VE comprises multiple tocopherol and tocotrienol isoforms with distinct biological properties; therefore, the present α‐tocopherol‐based structural findings should not be generalized to all VE isoforms without comparative analysis.

Overall, this study defines a hypothesis‐generating nutritional systems‐biology framework linking VE‐associated molecular programs to redox buffering, lipid‐inflammatory signaling, vascular response, and multicellular tissue remodeling in AIS. The SOD1/GCLC, PTGS1/PTGS2, and KDR‐centered axes provide testable molecular candidates for future biochemical, cellular, and clinically anchored validation.

## 5 Conclusion

In conclusion, this hypothesis‐generating analysis identifies database‐derived VE‐related molecular signatures that overlap with AIS‐associated redox, lipid‐inflammatory, and vascular‐response programs. The SOD1/GCLC, PTGS1/PTGS2, and KDR‐centered axes provide testable hypotheses for future validation of antioxidant buffering, prostaglandin‐related signaling, endothelial response, and multicellular tissue remodeling in AIS. Population‐level NHANES analyses further contextualized these molecular findings within adolescent clinical nutrition, but they did not establish an AIS‐specific vitamin E relationship. Future AIS cohorts integrating dietary vitamin E assessment, serum α‐ and γ‐tocopherol status with lipid adjustment, Cobb angle, curve progression, BMD, pubertal maturity, and oxidative/inflammatory biomarkers will be required to determine whether these molecular pathways vary with nutritional status, reflect compensatory responses, or represent downstream features of AIS tissue remodeling.

## Author Contributions

**Sitong Fang:** data curation, validation, investigation, writing – review and editing. **Zhizhong Geng:** data curation, methodology, software, validation, writing – review and editing, formal analysis. **Tianxiang He:** data curation, validation, investigation, writing – review and editing. **Jun Ren:** data curation, validation, investigation, writing – review and editing. **Ying Ma:** conceptualization, methodology, formal analysis, investigation, visualization, writing – original draft, software. **Shoujian Wang:** data curation, validation, investigation, writing – review and editing. **Lingjun Kong:** conceptualization, supervision, project administration, funding acquisition, resources, writing – review and editing. **Min Fang:** conceptualization, supervision, project administration, funding acquisition, resources, writing – review and editing. **Yuankun Han:** methodology, software, formal analysis, data curation, validation, writing – review and editing.

## Funding

This work was supported by the Shanghai Shenkang Hospital Development Center Emerging Frontier Technology Joint Research Project (SHDC12024110). National Natural Science Foundation of China (82374607). Shuguang Hospital Affiliated to Shanghai University of Traditional Chinese Medicine—Research Leadership Initiative (SGYYJBGS‐001). Shanghai Key Laboratory of Tuina Technique for Musculoskeletal Diseases (24dz2260200). National Administration of Traditional Chinese Medicine Pilot Project for TCM Intervention in Adolescent Idiopathic Scoliosis (Shanghai Huangpu District). Pudong New Area Science and Technology Commission Project (PKJ2023‐Y68); Health Science and Technology Project of the Pudong New Area Health Commission of Shanghai (PW2024D‐14).

## Ethics Statement

This study was based exclusively on publicly available, de‐identified datasets retrieved from the GEO database, NCBI BioProject/SRA, and the National Health and Nutrition Examination Survey (NHANES). No new human participants or animals were directly recruited or involved in this study, and no identifiable personal information was collected. Ethical approval and informed consent were addressed in the original studies where applicable. Therefore, additional institutional ethical approval was not required for this secondary analysis.

## Consent

Patient consent was not required for this study because it was based exclusively on publicly available, de‐identified datasets.

## Conflicts of Interest

The authors declare no conflicts of interest.

## Supporting information


**Data S1:** Complete differential miRNA expression and FunRich enrichment results for GSE235203.

**Data S2:** Complete differential mRNA results for GSE110359 and expression statistics for the prioritized genes.

**Data S3:** HERB compound mapping, VE–AIS shared‐target analyses, enrichment results, feature‐selection outputs, and exploratory ANN analysis.

**Data S4:** Complete KEGG‐ and GO‐GSEA results for SOD1, PTGS1, PTGS2, GCLC, and KDR.

**Data S5:** Single‐cell quality control, clustering, cell annotation, and feature‐gene localization analyses for PRJNA722100.

**Data S6:** Functional module scoring and bulk–single‐cell immune, stromal, and endothelial correlation analyses.

**Data S7:** CellChat ligand–receptor, signaling‐pathway, and concave–convex communication analyses.

**Data S8:** Molecular docking parameters and molecular‐dynamics simulation settings and results for α‐tocopherol and selected proteins.

**Data S9:** Exploratory AIS progression‐related miRNA contextual analysis using GSE286204.

**Data S10:** NHANES variable definitions, survey‐weighted analyses, full model outputs, and sensitivity analyses.

## Data Availability

The data that support the findings of this study were derived from publicly available resources. The plasma miRNA sequencing dataset GSE235203 and the bone marrow transcriptomic dataset GSE110359 are available from the Gene Expression Omnibus (GEO) database. The single‐cell transcriptomic dataset PRJNA722100 is available from the NGDC BioProject database. The Supporting Information generated for this study, including supplementary methods, extended results, tables, figures, and supporting analytical files, have been deposited in Zenodo and are publicly available at DOI: 10.5281/zenodo.20679528. No new individual‐level human data were generated in this study.
