# Supplementary material for: Vitamin E‐Related Molecular Signatures in Adolescent Idiopathic Scoliosis: A Hypothesis‐Generating Multi‐Omics Analysis
Source: Food Sci Nutr. 2026 Aug 6;14(8):e72190. doi: 10.1002/fsn3.72190 (PMC13447955; doi:10.1002/fsn3.72190)
Supplement: Supplementary file 1 [file FSN3-14-e72190-s005.docx]

Supplementary Material 9

AIS progression-related miRNA contextual mapping using GSE286204

*Supplementary Methods*

To provide AIS-specific progression-related molecular context that was unavailable in NHANES, an exploratory supplementary analysis was performed using GSE286204. This dataset contains circulating plasma miRNA profiles from AIS progressors, AIS non-progressors, and healthy controls. According to the GEO series description and accompanying metadata, AIS progressors were defined as patients who developed severe scoliosis at skeletal maturity, whereas non-progressors included patients with mild or moderate final curves.

The published progressor-associated upregulated miRNAs and the reported random forest miRNA panel were used as AIS progression-related circulating miRNA signatures. High-confidence predicted target genes for these miRNAs were obtained from miRDB-based target gene sets. The resulting miRNA target sets were intersected with the prioritized vitamin E-related AIS candidate genes and four manually curated biological modules corresponding to redox/glutathione buffering, arachidonic acid/prostaglandin-related signaling, VEGF/vascular response, and inflammatory signaling. Hypergeometric overlap tests were performed as exploratory analyses using 20,000 human protein-coding genes as the background.

Maximum baseline and final Cobb angles were parsed from the available GEO metadata when multiple curve values were reported for the same participant. In addition, the author-provided AIS progressor versus AIS non-progressor raw microarray matrix was reanalysed as a sensitivity check using Welch tests on log2-transformed intensity values. Spearman correlations between the published progressor-associated miRNAs and parsed maximum baseline/final Cobb angles were also calculated as exploratory analyses.

This supplementary analysis was intended to provide AIS progression-related molecular context only. It was not designed to test whether vitamin E exposure or status was associated with Cobb angle or curve progression, because GSE286204 does not include dietary vitamin E intake, serum α-tocopherol, serum γ-tocopherol, lipid-adjusted tocopherol status, or oxidative stress biomarkers.

*Supplementary Results*

GSE286204 included AIS progressors, AIS non-progressors, and healthy controls in the discovery microarray cohort. Parsed Cobb angle metadata confirmed the expected clinical severity contrast between AIS progressors and non-progressors. Non-progressors showed lower baseline and final Cobb angles, whereas progressors showed higher final Cobb severity at skeletal maturity.

Target mapping of the published AIS progressor-associated miRNA signature produced a broad set of predicted target genes. Descriptive overlap analysis showed that these miRNA target sets intersected with the biological modules proposed in the main study. Overlapping genes included GCLC and GCLM in the redox/glutathione module, PTGES3 in the prostaglandin-related module, NRP1, NRP2, TEK, and VEGFA in the VEGF/vascular-response module, and CCL2 and MAPK1 in the inflammatory signaling module. The reported random forest miRNA panel showed a similar descriptive overlap pattern involving GCLC, PTGES3, NRP1, TEK, CCL2, and MAPK1.

At the individual miRNA level, several progressor-associated miRNAs were predicted to target genes within the proposed redox–lipid inflammatory–vascular framework. For example, miR-1-3p was predicted to target GCLC, NRP1, and CCL2; miR-148b-3p was predicted to target PTGES3, NRP1, and TEK; miR-19a-3p and miR-19b-3p were predicted to target MAPK1; and miR-576-5p was predicted to target GCLM and VEGFA.

Exploratory hypergeometric tests did not show FDR-significant module enrichment. In the raw-matrix sensitivity analysis, several miRNAs showed nominal differences between AIS progressors and non-progressors, but none remained significant after Benjamini–Hochberg correction. Similarly, no individual miRNA-Cobb angle correlation remained FDR-significant. Therefore, the GSE286204 analysis was interpreted as descriptive AIS progression-related molecular context rather than independent statistical validation of the vitamin E-related framework.

Supplementary Table S9.1. Descriptive overlap between progressor-associated miRNA target sets and proposed biological modules

| miRNA signature | Module | Overlapping genes |
| --- | --- | --- |
| 15 progressor-associated miRNAs | Redox/glutathione | GCLC, GCLM |
| 15 progressor-associated miRNAs | Prostaglandin-related | PTGES3 |
| 15 progressor-associated miRNAs | VEGF/vascular response | NRP1, NRP2, TEK, VEGFA |
| 15 progressor-associated miRNAs | Inflammatory signaling | CCL2, MAPK1 |
| 6-miRNA random forest panel | Redox/prostaglandin/vascular/inflammatory modules | GCLC, PTGES3, NRP1, TEK, CCL2, MAPK1 |

*Scope and interpretation of exploratory analysis*

The GSE286204 supplementary analysis partially addresses the absence of Cobb angle and progression information in NHANES by introducing an AIS-specific public dataset with progression-related clinical context. The descriptive overlap between progressor-associated miRNA targets and redox/glutathione, prostaglandin-related, inflammatory, and vascular-response modules is consistent with the biological framework proposed in the main manuscript.

However, this analysis does not provide vitamin E-specific clinical validation. GSE286204 does not contain dietary vitamin E intake, serum tocopherol measurements, lipid-adjusted vitamin E status, or direct oxidative stress biomarkers. Furthermore, the overlap analyses, raw-matrix reanalysis, and miRNA-Cobb correlation analyses did not yield FDR-significant results. Therefore, these findings should be interpreted only as exploratory molecular context linking AIS progression-associated circulating miRNA signatures to the same biological modules highlighted in the main study. They should not be interpreted as evidence that vitamin E status is associated with Cobb angle, curve progression, or AIS severity.
